# Increased/Targeted Brain (Pro)Drug Delivery via Utilization of Solute Carriers (SLCs)

**DOI:** 10.3390/pharmaceutics14061234

**Published:** 2022-06-10

**Authors:** Johanna Huttunen, Santosh Kumar Adla, Magdalena Markowicz-Piasecka, Kristiina M. Huttunen

**Affiliations:** 1 School of Pharmacy, Faculty of Health Sciences, University of Eastern Finland, P.O. Box 1627, FI-70211 Kuopio, Finland; johanna.huttunen@uef.fi (J.H.); santosh.adla@uef.fi (S.K.A.); 2 Institute of Organic Chemistry and Biochemistry (IOCB), Czech Academy of Sciences, Flemingovo Namesti 542/2, 160 00 Prague, Czech Republic; 3 Department of Pharmaceutical Chemistry, Drug Analysis and Radiopharmacy, Medical University of Lodz, ul. Muszyńskiego 1, 90-151 Lodz, Poland; magdalena.markowicz@umed.lodz.pl

**Keywords:** blood–brain barrier (BBB), brain drug delivery, prodrugs, solute carriers (SLCs)

## Abstract

Membrane transporters have a crucial role in compounds’ brain drug delivery. They allow not only the penetration of a wide variety of different compounds to cross the endothelial cells of the blood–brain barrier (BBB), but also the accumulation of them into the brain parenchymal cells. Solute carriers (SLCs), with nearly 500 family members, are the largest group of membrane transporters. Unfortunately, not all SLCs are fully characterized and used in rational drug design. However, if the structural features for transporter interactions (binding and translocation) are known, a prodrug approach can be utilized to temporarily change the pharmacokinetics and brain delivery properties of almost any compound. In this review, main transporter subtypes that are participating in brain drug disposition or have been used to improve brain drug delivery across the BBB via the prodrug approach, are introduced. Moreover, the ability of selected transporters to be utilized in intrabrain drug delivery is discussed. Thus, this comprehensive review will give insights into the methods, such as computational drug design, that should be utilized more effectively to understand the detailed transport mechanisms. Moreover, factors, such as transporter expression modulation pathways in diseases that should be taken into account in rational (pro)drug development, are considered to achieve successful clinical applications in the future.

## 1. Introduction

Diseases of the central nervous system (CNS), including neurodegenerative and neurodevelopmental diseases, are one of the greatest threats to public health. According to the World Health Organization, these diseases account for 12% of deaths worldwide and the economic burden of direct and indirect healthcare costs are substantial [1]. To make matters worse, these numbers are expected to increase as the population ages [2]. Most brain diseases lack effective drug therapies, since the brain is protected by multiple mechanisms that block drugs from entering and reaching their target sites [3,4]. Firstly, the majority of drugs are unable to cross the blood–brain barrier (BBB), which is made of capillary endothelial cells that are connected very closely by tight junctions. The endothelial cells of the BBB are also metabolically very active in protecting the brain tissue from various xenobiotics and microbes and effluxing them back to the bloodstream at the luminal membranes of the BBB via numerous ATP-binding cassette (ABC) transporters, including P-glycoprotein (P-gp, *ABCB1*), multidrug resistance proteins 2 (MRP2, *ABCC2*) and 4 (MRP4, *ABCC4*), and breast cancer resistance protein (BCRP, *ABCG2*) [5]. It has been estimated that 98% of all current drugs do not cross the BBB at sufficiently high amounts to therapeutically treat CNS diseases [6]. However, the brain drug disposition is highly regulated not only by the BBB but also by neurons and glial cells that can serve as a secondary barrier to brain drug exposure [7,8]. Surprisingly, the inability of drugs to cross the cellular membranes of brain parenchymal cells has been less studied in the past, although many of the novel CNS targets are intracellular proteins.

Endogenous solute carriers (SLCs) transport essential substances and xenobiotics across the cell membranes and, e.g., across the BBB but also into the brain parenchyma. In the brain, their primary role is to regulate the supply of essential nutrients, such as amino acids, sugars, vitamins, nucleosides, and electrolytes for the endothelial and parenchymal cells. However, transporters can also carry various drugs and toxins, and thus, they are major determinants of CNS drug/toxin exposure [6,8]. Although SLCs, nearly 500 transporters overall, present promising drug carriers as well as drug targets in the brain, they are still poorly characterized and utilized in rational drug research and development today [9,10]. So far, the expression and function of many SLCs in brain microvascular endothelial cells have been extensively described [5]; however, less is known about SLCs’ roles in drug disposition, response, and drug–drug interactions in the parenchymal cells. Nevertheless, carrier-mediated transport via SLCs to improve brain drug disposition can be achieved, if structural features required for the interactions with the selected transporter are known. Unfortunately, the recent focus has been paid to only a selected number of transporters.

Prodrugs are compounds with little or no pharmacological activity of their own. They are designed to be bioconverted to active drugs either chemically or enzymatically, which releases the active parent drug and the promoiety [11,12]. The prodrug approach is used to overcome pharmaceutical and/or pharmacokinetic limitations that are preventing the successful clinical use of the parent drugs. Moreover, by creating transporters’ substrate mimicking prodrugs, improved or even targeted drug delivery resulting in enhanced clinical outcomes, can be achieved. This is often the most feasible method to retain the pharmacological potency of a potential drug candidate while changing its structural features into ones that can improve the drug delivery to the target site. Curiously, it has been estimated that approximately 10% of all worldwide approved drugs are currently prodrugs and 11% of new small molecular entities approved by the Food and Drug Administration (FDA) during the years 2008–2018 were prodrugs [13].

However, successful prodrug design and development requires a deeper understanding of the structure and transport mechanisms, which is a dynamic process with conformational changes [14,15]. To date, these so-called “moving barriers” are better understood due to the invasion of cryo-electron microscopic (cryo-EM) structural analyses of different conformations of these proteins. Moreover, improved computational power and more sophisticated methods, such as molecular dynamics simulations (MDS), have enabled us to put these conformational changes in order and describe the detailed mechanisms of the transport process [16,17]. In this comprehensive review, the expression and major functions of several known brain drug carriers belonging to the SLC family are presented. Moreover, their possible utilization for the brain (pro)drug delivery and intrabrain drug targeting are discussed. Most importantly, the effects of transport mechanisms aiding in (pro)drug design and the significance of the transporters’ expression/function changes in specific diseases are considered.

## 2. Glutamate and Neutral Amino Acid Transporter Family (SLC1A)

**Figure 1:**
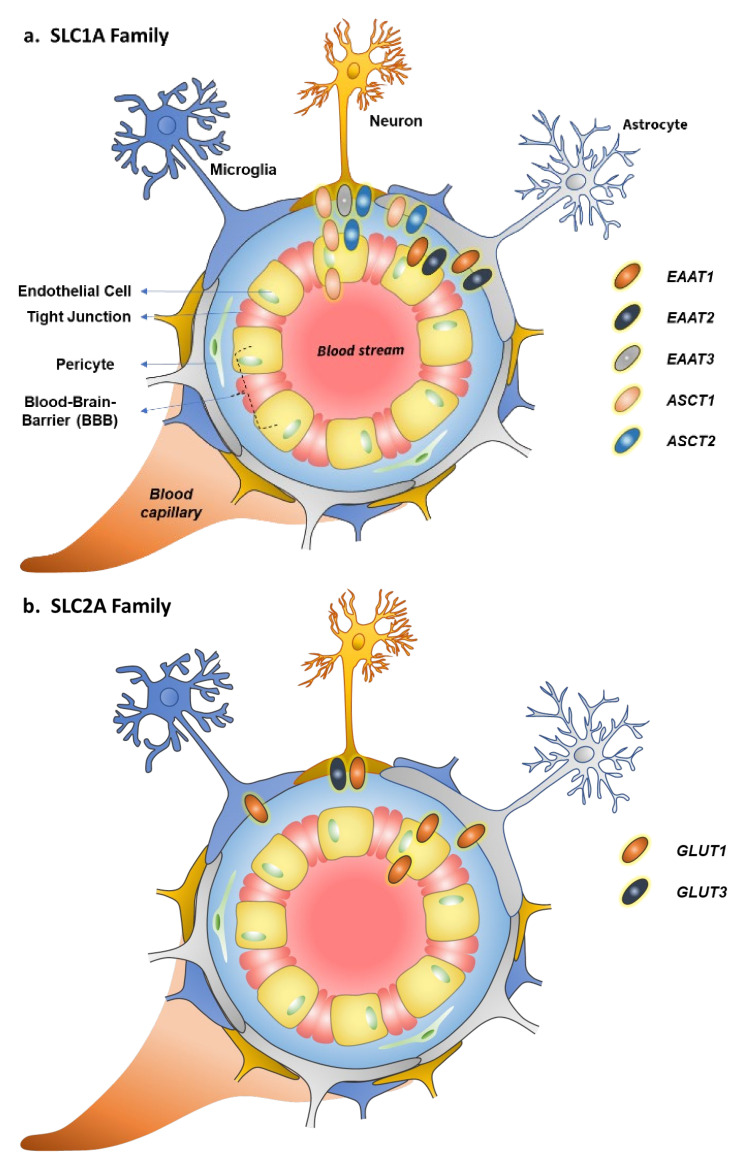

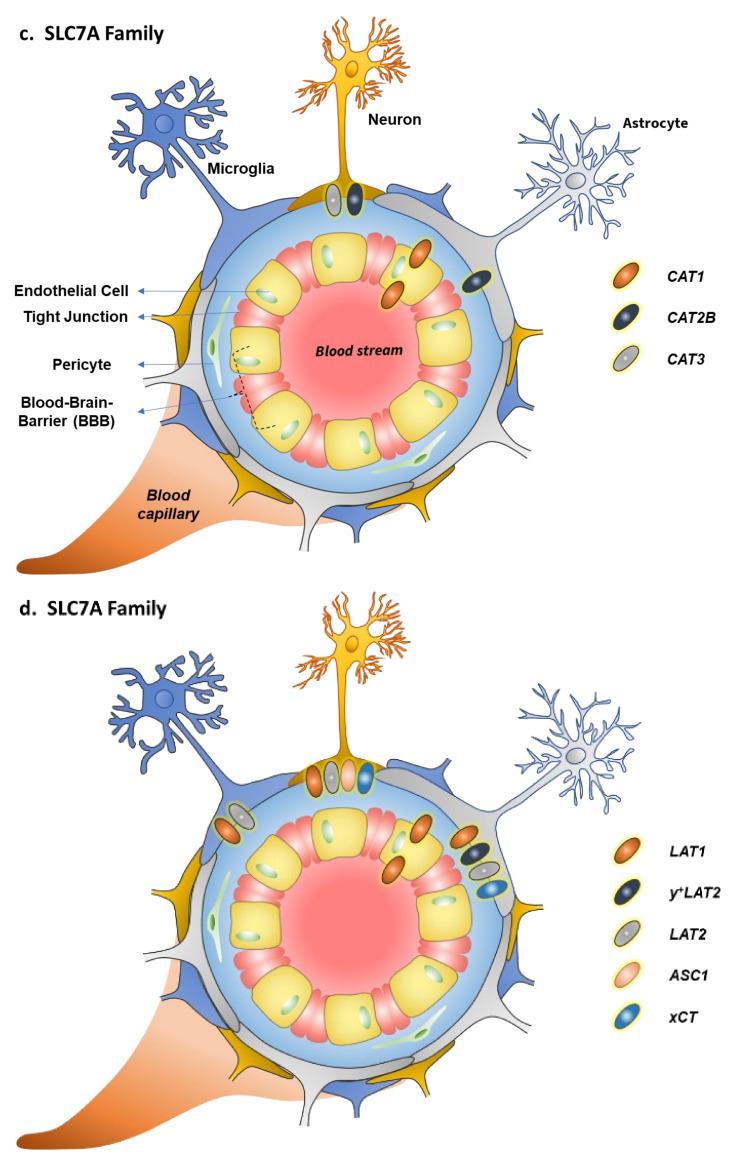

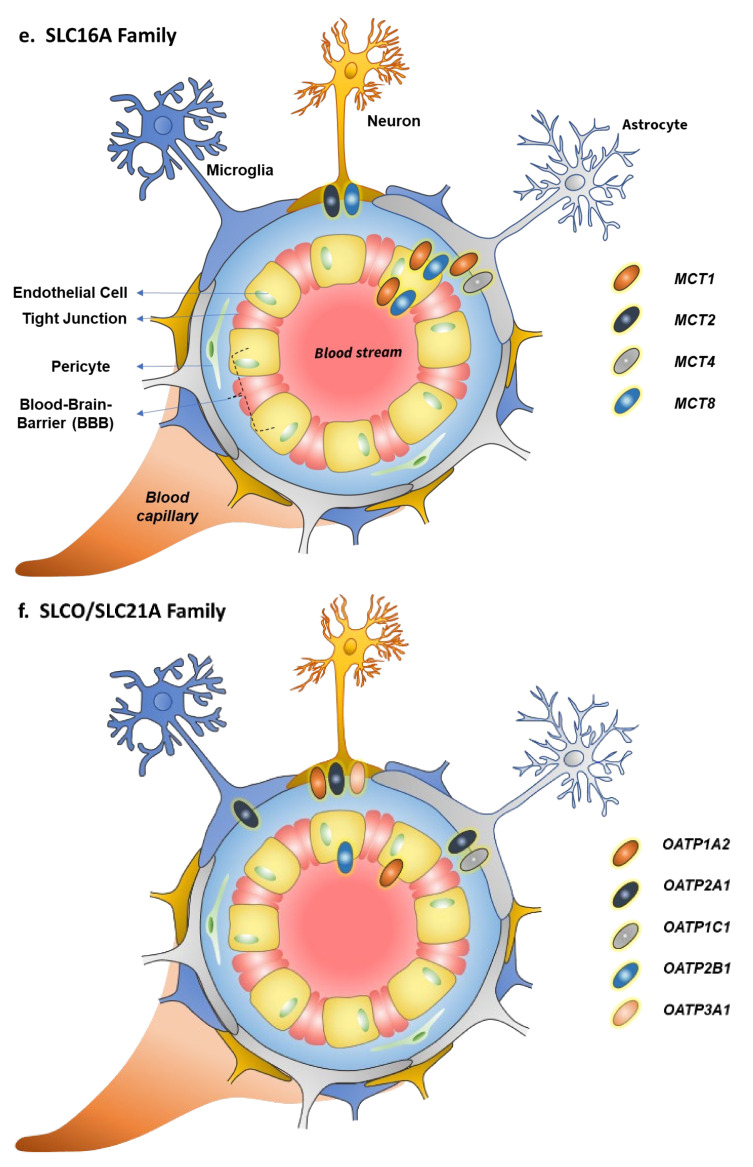

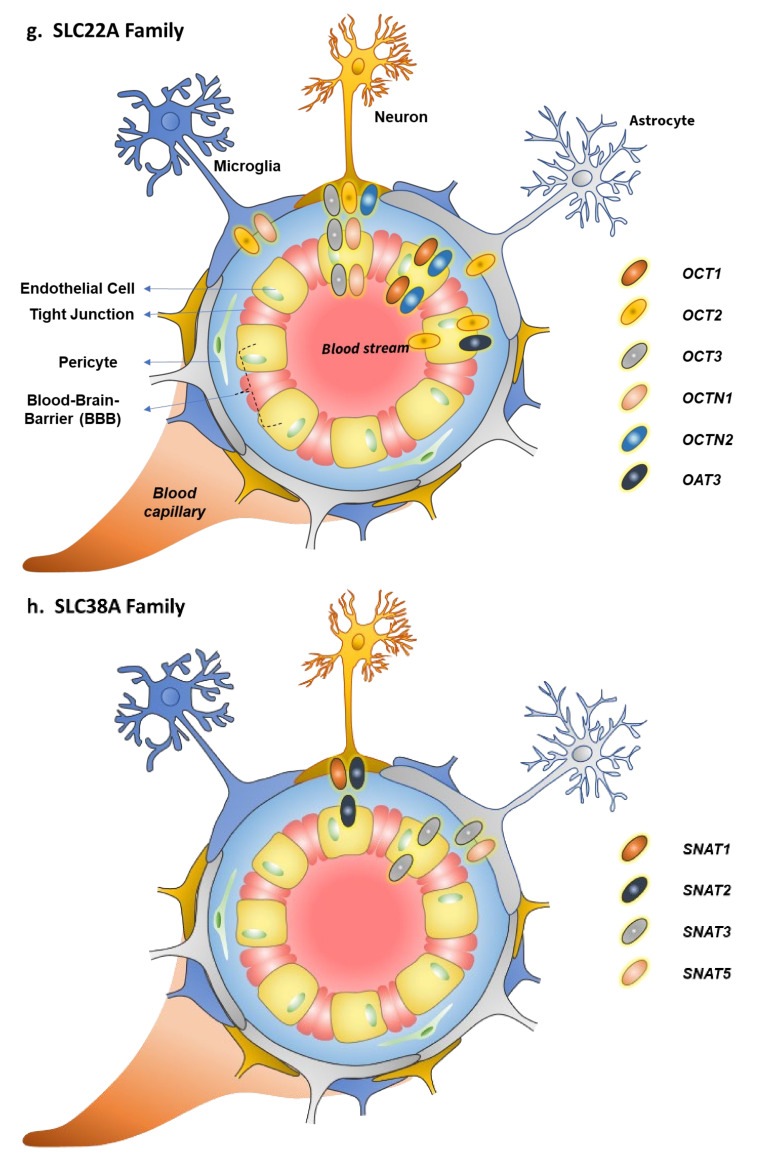
Neuro-glia vascular units illustrating transporter expression/localization of (**a**) of SLC1A-, (**b**) SLC2A-, (**c**,**d**) SLC7A-, (**e**) SLC16A-, (**f**) SLC21A-, (**g**) SLC22A-, and (**h**) SLC38A-families in different cell types. The transporters are illustrated with round-shaped objects with different colors.

The SLC1A family consists of seven members divided into two groups, namely, excitatory amino acid transporters (EAATs) and alanine, serine, and cystine transporters (ASCT1 and 2). EAATs are high-affinity L-glutamate (and L-aspartate) transporters, while ASCTs facilitate the transfer of several neutral amino acids across the cell membranes (Table 1). EAATs are mainly localized in the brain, particularly in glutamatergic synapses, where they have a fundamental role in maintaining normal glutamate homeostasis [18]. In addition to the abluminal side of the BBB, EAAT1 and 2 (*SLC1A3* and *SLC1A2*) are mainly localized in glial cells, while EAAT3 (*SLC1A1*) is mainly expressed in neurons (Figure 1a) [5,19,20,21]. Other members of this family, EAAT4 and 5 (*SLC1A6* and *SLC1A7*), are also found in the Purkinje cells of the cerebellum and retina, respectively; however, their function in glutamate-gated chloride conductance (stronger with EAAT4–5 compared to EAAT1–3) is not well understood today [18]. L-Glutamate uptake via EAATs requires simultaneous cotransport of three Na^+^ ions and countertransport of one K^+^ ion across the cell membrane. The K^+^ countertransport has been proposed to be an independent step of the L-glutamate translocation process.

**Figure 2:**
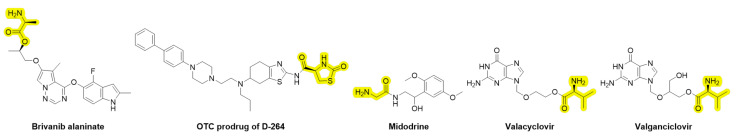
Molecular structures of proposed SLC1A prodrugs with the promoieties highlighted with yellow color.

Due to the relevance of EAATs in excitatory neurotransmission, they are the key players in preventing neurons from glutamate excitotoxicity induced by pathological conditions and injuries such as neurological disorders and stroke [18,22]. Moreover, EAATs have been acknowledged to have a major role in antioxidant defense balance, since L-glutamate is a precursor for the endogenous antioxidant, tripeptide glutathione (GSH). Since the expression of EAATs in the brain is downregulated in many CNS diseases, including epilepsy, Alzheimer’s disease (AD), Parkinson’s disease (PD), Huntington’s disease (HD), amyotrophic lateral sclerosis (ALS), and ischemic stroke to name a few, the endogenous regulation of their expression levels is extensively studied (Table 1). For example, EAAT1 and 2 are upregulated via adenylate cyclase-activating polypeptide (PACAP), transforming growth factor α (TGFα), epidermal growth factor (EGF), estrogen and estrogen receptor modulators tamoxifen and raloxifene [22]. Some drugs, such as β-lactam antibiotic ceftriaxone have also been reported to upregulate EAAT2 expression via stimulated nuclear translocation of p65 and initiation of the transcription nuclear factor κB (NF-κB) [23,24], while amphetamine is known to modulate EAAT3 plasma membrane expression via induced endocytosis in dopamine neurons [25]. Although EAATs have not been mainly considered drug carriers, several selective inhibitors of EAAT1, EAAT2, and EAAT3 have been reported (Table 1) [26,27,28]. Furthermore, it has been speculated if L-cystine derivatives, like L-2-oxothiazolidine-4-carboxylic acid (OTC), could have interactions with EAATs and, thus, be used as selective promoieties to carry parent drugs in a prodrug form, like in the case of D-264, a neuroprotective agent that has been studied in the treatment of PD (Figure 2) [29].

Although ASCT1 (*SLC1A4*) and ASCT2 (*SLC1A5*) are ubiquitously expressed throughout the body, they are also found in the brain; mainly in neurons and astrocytes, but also to some extent at the BBB (Table 1) [5,30,31,32,33,34]. Both ASCT1s mediate sodium- and pH-dependent transport of several neutral amino acid substrates, not only L-alanine, L-serine, and L-cystine (ASCT1 only), but also L-glycine, L-methionine, L-valine, L-leucine, L-isoleucine, and L-threonine. Bidirectionally, they can also mediate the efflux of glutamate (ASCT1) or glutamine (ASCT2) out of the cells. Mutations in ASCT1 have been associated with neurodevelopmental deficits, such as alterations in motor function, spatial learning, and affective behavior [35], while expression levels of ASCT2 have been reported to be increased in several cancers, including brain, colon, pancreas, liver, and lung cancer [36,37]. Therefore, ASCT2 has been widely studied as a pharmacological target to inhibit cancer cell growth and development, although ASCT2 expression modulation could also be utilized in neurodevelopmental disorders.

It would be tempting to think that amino acid conjugates, like those attached to L-alanine, would serve as potential ASCT-utilizing prodrugs. However, as proved by several prodrugs, including tumorigenic drug brivanib alaninate (BMS-582664; a selective dual inhibitor of vascular endothelial growth factor receptor 2 (VEGFR-2) and fibroblast growth factor receptor 1 (FGFR-1)), vasopressor/antihypotensive agent midodrine (ProAmatine^®^, Gutron^®^; a glycine prodrug of desglymidodrine (DMAE), a selective α1-receptor agonist), and antiviral agents valacyclovir (Valtrex^®^) and valganciclovir (Valcyte^®^) (Figure 2), their oral absorption is mediated via either intestinal proton-coupled peptide transporter 1 (PepT1, *SLC15A1*) or sodium-dependent neutral amino acid transporter (ATB^0,+^, *SLC6A14*) [38]. However, the interactions and brain exposure via ASCTs of these compounds have not been well studied in the past, mainly since all these prodrugs are relatively rapidly bioconverted during the first-pass metabolism by ubiquitous hydrolyzing enzymes. Moreover, all these exemplary prodrugs have been attached to the amino acids via the carboxylic acid group, which may be required to be left untouched and the parent drugs should be attached to the side chain of the amino acids to attain sufficient interactions with ASCTs. Therefore, more detailed structural studies are warranted to develop successful brain-delivered ASCT-utilizing compounds.

**Figure 3:**
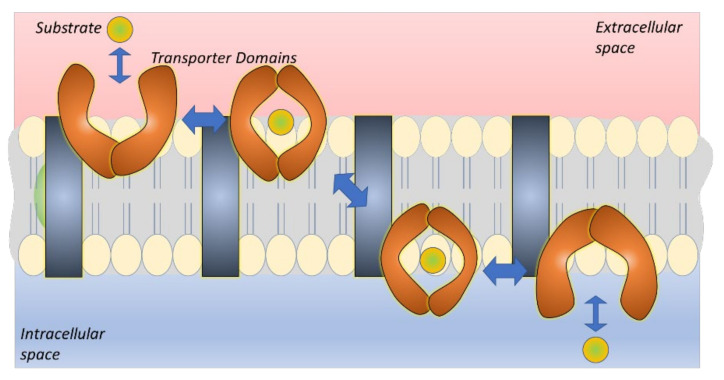
The elevator-type transport mechanism of EAATs and ASCTs. The substrate (green ball-shape) is bound to the transporter on the extracellular side of the plasma membrane. The conformational movements of the transporting domains (orange banana-shapes) close the “gate”, which is followed by vertical translocation of this complex in relation to the static domain (blue). Finally, the second conformational movement opens the “gate” and releases the substrate into the cytosolic side.

ASCT1 and 2 share 58% sequence identity and together with EAATs the whole family shares only 21% sequence identity [39]. Both EAATs and ASCTs are formed from eight transmembrane helices, and their amino acid transport mechanism is well understood. Curiously, all SLC1A family members function with a one-gate elevator mechanism, in which the substrate is occluded in the transport domain that subsequently performs a large movement from the extracellular site to the intracellular site (Figure 3). The binding sites of ASCT1 and 2 are relatively well known today and they differ from each other [40,41]. For example, ASCT1 recognizes also D-amino acids and it has been proposed to have a major role in the brain exposure of D-serine, *N*-methyl-D-aspartate (NMDA) receptor co-agonist (Table 1), thereby, remarkably affecting the brain disorders, such as schizophrenia [35,42]. Although ASCT1 has not been utilized as a drug carrier for a rational drug design, a lot of interest has been laid in ASCT2, particularly, in the development of selective ASCT2 inhibitors, as mentioned above. From these, *O*-benzyl-L-serine and *S*-benzyl-cysteine are well-known examples [43]. Later on, other amino acid analogs, such as proline-, serine-, and glutamine-mimetics have also been developed as ASCT2-selective inhibitors [44,45,46]. Both ASCT1 and 2 have also been noticed to be inhibited by phenylglycine analogs [42]. However, as with ASCT1, it would be great to see in the future if ASCT2 could be utilized more extensively as a (pro)drug carrier, e.g., in cancer-targeted applications. Nevertheless, the elevator mechanisms may be the determinant of the drug delivery efficiency and exclude the transport of larger compounds. Hense, other SLCs with different mechanisms may be more feasible (pro)drug carriers for brain-targeted purposes than the presented SLC1A-family members.

## 3. Glucose Transporter Family (SLC2A)

Glucose transporters provide basal glucose levels for energy production in many tissues and cell types, from red blood cells, muscles, and adipose tissue to the brain and neurons. The main glucose transporters in the brain are glucose transporter 1 (GLUT1, *SLC2A1*) and 3 (GLUT3, *SLC2A3*) (Table 2) [5,47]. Ubiquitously localized GLUT1 has been found at the BBB in heavily glycosylated form (55 kDa), both at the luminal and abluminal sides of the endothelial cells. A less glucosylated form (45 kDa) is in turn found in the astrocytes and to a lesser extent also in the neurons and microglia (Figure 1b). Contrarily, GLUT3 has been historically called “a neuronal glucose transporter”, as it is almost exclusively expressed in the neurons. In addition, among the classified 14 glucose transporters, GLUT2 and GLUT4–8 have been identified from the brain, either from the BBB or parenchymal cells, although with much smaller expression levels than GLUT1 and 3 [47]. Moreover, their physiological roles in the brain are not yet fully understood.

The preferred substrates for both GLUT1 and GLUT3 are hexoses and pentoses, in pyranose conformation, such as D-glucose, D-galactose, and D-mannose (Table 2). They also carry glucose analogs such as 2-deoxy-D-glucose, and their transport is described as high affinity–high capacity. GLUT1 transports specifically glucosamine and dehydroascorbic acid (vitamin C), as well as 2-deoxy-2-[^18^F]-D-glucose that has been used as a radiolabeled marker in positron emission tomography (PET) for the diagnosis and monitoring of different diseases [48]. GLUT1/3-mediated transport is inhibited by cytochalasin B, forskolin, phloretin, and other flavonoids. GLUT1 can also be selectively inhibited by WZB117, BAY-876, STF-31, fasentin, apigenin, while glycogen synthase kinase-3 (GSK-3) inhibitors have been reported to selectively inhibit GLUT3 [49].

Overexpressions of GLUT1 and 3 have been found with a variety of human carcinomas and this phenomenon has been associated with aggressiveness and poor survival rate (Table 2) [50,51,52]. The phosphatidylinositol 3-kinase (PI3K)-Akt pathway, hypoxia-inducible factor-1 (HIF-1), and p53 are known to upregulate both GLUT1 and 3 on a transcriptional level, while Ras and c-Myc oncogenes have been associated only to GLUT1 upregulation [52]. In addition, hypoglycemia (low blood glucose caused by starvation, liver/kidney diseases, or infections) and hypoxic condition related to cerebral ischemia can also upregulate brain GLUT1 and GLUT3 expressions [47]. In turn, in hyperglycemia (high blood glucose due to untreated diabetes or pancreatitis) as well as at the early onset of AD have been demonstrated to downregulate the GLUT1 and GLUT3 levels in the brain. Moreover, mutations of GLUT1 that are inherited in an autosomal dominant or autosomal recessive manner causes GLUT1 deficiency syndrome (G1DS) with severe effects on neural functions and brain development. Curiously, several drugs and alcohol consumption have been associated with brain expression or activity modulation of GLUT1/3. Therefore, drug treatments that are related to these GLUTs or utilize GLUTs for their delivery should be carefully and profoundly evaluated during the preclinical phase. However, GLUT expression modulation can also be an important therapeutic target, e.g., in AD, GLUT1/3 upregulation may prevent the disease progression, and in stroke, GLUT1/3 upregulating treatment may have the potential to improve the final outcome faster. As a cancer treatment, selective GLUT1/3 downregulation could be used to inhibit increased glycolysis and, thus, cell growth.

**Figure 4:**
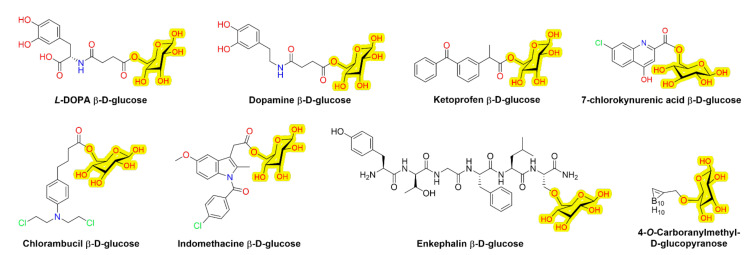
Molecular structures of GLUT1-utilizing prodrugs with promoieties highlighted with yellow color.

From all glucose transporters, GLUT1 has been mostly utilized for brain-targeted drug delivery by a prodrug approach and the first brain-targeted GLUT1-utilizing prodrugs were reported already 20 years ago. These include, for example, β-D-glucosyl and β-D-galactosyl derivatives of 7-chlorokynurenic acid to improve NMDA receptor-mediated seizures, and β-D-glycosyl derivatives of dopamine and L-dopa to improve reserpine- or morphine-induced locomotion (Figure 4) [53,54,55]. In addition, β-D-glucose has also been attached to alkylating agent, chlorambucil to improve delivery into brain tumors, and to neuroactive peptide, enkephalin to improve analgesic effects [56,57]. Later on, the β-D-glucosyl promoiety has also been used with non-steroidal anti-inflammatory drugs (NSAIDs), and at least GLUT1-mediated brain uptake of ketoprofen and indomethacin prodrugs has been reported [58]. However, the benefits of the approach to improve clinical outcomes have remained unclear so far. Interestingly, novel brain-targeted GLUT1-utilizing prodrugs have not been reported recently, despite their early discovery. Instead, the approach has been used more extensively for cancer-targeting purposes.

Notably, most of the reported glyco-prodrugs are esters and relatively unstable during the first-pass metabolism, which most likely limits their use for brain-targeting purposes. Many of the glucose, galactose, or glucuronic acid prodrugs are bioconverted by either β-glucosidase, β-galactosidase, or β-glucuronidase, respectively. These enzymes are relatively ubiquitously expressed in peripheral tissues as well as in the brain, and curiously, they are also over-expressed in many different types of cancer cells [59,60]. There are also a few examples of more stable amide and ether prodrug bond-containing glyco-derivatives (chlorambucil and enkephalin derivatives) [56,57]. However, these compounds may be enzymatically too stable in the brain and, thus, they may require to be active on their own, without releasing the active parent drug. Thus, utilizing GLUTs for brain-targeting purposes may be more suitable for compounds that are not prodrugs and, therefore, do not require the bioconversion step. A great example is brain tumor-targeted boron (^10^B) compounds (Figure 4) used in the boron neutron capture therapy (BNCT), in which a low energy thermal neutron beam initiates a fission reaction of ^10^B that is selectively accumulated into the cancer cells via GLUT1. This produces high-energy α-particles (^4^He) and ^7^Li atoms that ultimately destroy the cancer cells without affecting non-boron-containing healthy cells [61,62,63].

**Figure 5:**
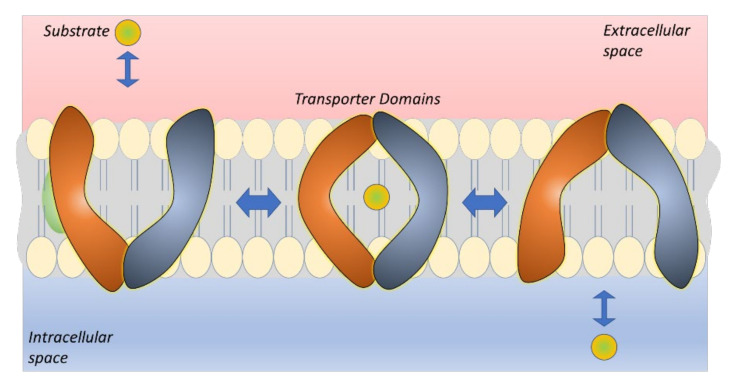
The rocker switch transport mechanism of GLUT1 and 3. The substrate (green ball-shape) is bound to the V-shaped transporter (outward-open state) on the extracellular side of the plasma membrane. The conformational movements of the transporter domains (orange and blue banana-shapes) trigger outward-occluded and inward-occluded states (only one state is showing). Finally, the substrate is released from V-shaped inward-open conformation to the cytosolic side.

The glucose transporters are sodium-independent bidirectional and they commonly have 12 putative transmembrane-spanning α-helices and a single site for *N*-linked glycosylation. The transport mechanism of GLUT1 and GLUT3 is well known and they follow a model called a “rocker switch”, This mechanism has four distinct states; (1) outward-open state, in which the ligand binds to the transporter causing the outer gate to close, (2) outward-occluded state, in which a rocker-switch takes place forming (3) inward-occluded state that is followed by the opening of the inner gate, and finally (4) inward-open state, from which the ligand is released (Figure 5) [14,15,64]. Today, it is well known which interactions can result in conformational changes in GLUT1 and, thus, induce the translocation of glucose derivatives in the cavity [65,66,67]. Thus, molecular modeling can be a really helpful tool for designing GLUT1 substrates that are truly transported through the protein cavity and not only bind to the protein on the plasma membrane. However, it would be great to see in the future if this is applicable also for GLUT3 and if neuron-targeted drug delivery can be achieved by utilizing GLUT3.

## 4. Cationic and Neutral Amino Acid Transporter Family (SLC7A)

The SLC7A family contains cationic amino acid transporters (CATs) and heterodimeric amino acid transporters (HATs). CATs are relatively ubiquitously localized in the body; however, in the brain CAT1 (*SLC7A1*) is mainly found at the luminal and abluminal sides of the BBB [5,68], while, CAT3 (*SLC7A3*) has been identified as a neuron-specific transporter (Table 3, Figure 1c) [69]. CAT2 (*SLC7A2*) has been found in two splicing variants, CAT2A, low affinity, not localized in the brain, and CAT2B, high affinity, localized in neurons and oligodendrocytes and induced in astrocytes [70,71]. In addition to CAT1–3, CAT4 (*SLC7A4*) and an orphan *SLC7A14* have been identified from the brain, although their functions and roles as carriers are not well characterized yet.

According to their name, the primary substrates of CATs are cationic amino acids, such as L-arginine, L-lysine, and L-ornithine, which are transported in a proton-coupled, sodium-independent manner (Table 3). Since L-arginine is a precursor for both L-ornithine and nitric oxide (NO) synthesis, CATs have a crucial role in regulating different homeostatic and proliferating actions in the brain (and in the peripheral tissues) [72]. Curiously, it has been reported that the expression of CATs is downregulated by NMDA receptor activation and they regulate neuronal processes via the mammalian target of rapamycin (mTOR) pathway [73]. In turn, CAT1 has been found to promote cell growth, proliferation, and metastasis, and it is upregulated in colorectal and breast cancers as well as in hepatitis B virus-induced hepatocellular carcinoma and lymphocytic leukemia [74,75,76,77]. In addition, CAT2A and 2B have been detected in different breast cancer cell lines.

The transport mechanism of CATs has not yet been resolved; however, some mechanistic insights and differences to HATs have been recognized [78]. To date, CATs have been recognized neither as potential drug carriers nor drug targets, and therefore, rationally developed substrates or inhibitors have not been reported yet (Table 3). However, CAT1 could be harnessed for increased brain drug delivery and together with CAT3-utilizing compounds, neuronal targeting could be achieved. As an example, L-arginine, L-lysine, and L-ornithine could be utilized as promoieties in the prodrug design. However, more details on the interactions between these promoieties and CATs are required in order to attach the parent drug to the correct functional group of the amino acid and, thus, to prepare successful prodrug candidates.

HATs consist of two subunits: a heavy subunit from the SLC3A family and a light subunit from the SLC7A family that are linked together via a disulfide bond. Two members of SLC3A family are rBAT (*SLC3A1*) and 4F2hc (*SLC3A2*) and these heavy subunits are *N*-glycosylated [79]. Their primary role is more regulatory, e.g., they traffic the holotransporter to the plasma membrane. However, recently there has been some evidence that heavy subunits may also participate in the ligand recognition process. Furthermore, 4F2hc has been found to mediate integrin-dependent tumorigenesis, and therefore, it is also overexpressed in some types of cancers [80,81]. Contrarily, several mutations in rBAT are found to affect the b^0,+^ transport, causing cystinuria. Nevertheless, the light subunits catalyze the transport function of HATs. L-Type amino acid transporter 1 (LAT1; *SLC7A5*) is probably the most studied transporter of the SLC7A-family in brain delivery applications, although other members, including y+L-type amino acid transporter 2 (y+LAT2; *SLC7A6*), L-type amino acid transporter 2 (LAT2; *SLC7A8*), alanine–serine–cysteine transporter 1 (Asc-1; *SLC7A10*), and cystine/glutamate transporter (xCT, *SLC7A11*), are also expressed in the brain and could be utilized in the brain drug delivery (Table 3).

LAT1 coupled to 4F2hc is a sodium- and pH-independent transporter carrying large, neutral, aromatic, or branched L-amino acids (L-leucine, L-phenylalanine, L-tyrosine, L-tryptophan, L-histidine, L-methionine, and L-valine) into the cells [82,83]. LAT1 is distributed throughout the body, and it is highly expressed in tissues that require a high amino acid supply, such as the brain, placenta, and bone marrow [84,85]. In the brain, LAT1 is localized not only at the luminal and abluminal membranes of BBB, but also in the parenchymal cells, neurons astrocytes, and microglia (Figure 1d) [5,86,87]. Moreover, LAT1 is upregulated in a variety of cancers and their metastases [88,89,90]. High LAT1 expression has been associated with significantly shorter survival of patients and poorer prognosis of breast and prostate cancers [91,92], and with metastasis of different cancer cell types [93]. The regulation mechanisms are still not well understood; however, hypoxia and HIF-2α, c-MYC, and RAS-MEK-ERK pathways have been proposed to be involved [90]. Curiously, mutations of LAT1 at the BBB have been associated with decreased LAT1 function in autism spectrum disorders (ASD) [94]. Thus, LAT1 expression modulation could be a potential target in neurodevelopmental diseases, such as ASD.

**Figure 6:**
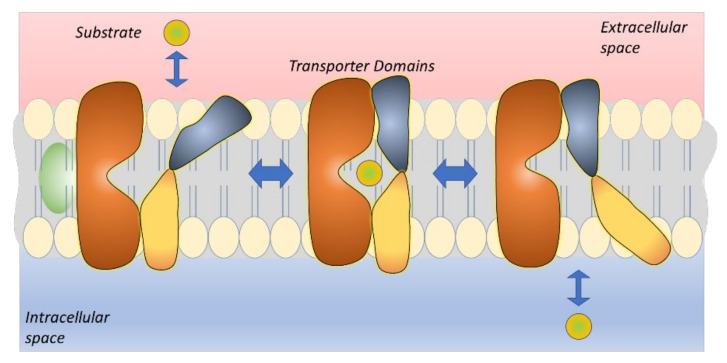
The rocking bundle transport mechanism of LAT1. The substrate (green ball-shape) is bound to the K-shaped transporter (outward-open state) on the extracellular side of the plasma membrane. The subsequent conformational movements of the transporter domains (first blue and then yellow shapes) result in the release of the substrate at the cytosolic side (inward-open state).

LAT1 catalyzes the transport of the thyroid hormones triiodothyronine (T3) and thyroxine (T4), but also amino-acid-mimicking drugs, such as the antiparkinsonian drug L-dopa, anti-cancer agent melphalan, muscle relaxer baclofen, and anticonvulsants gabapentin and pregabalin (Table 3). Due to the intensive research, the binding and translocation of ligands through LAT1 is relatively well known today [95,96,97,98]. The definite structural requirements include the presence of amino and carboxylic acid functional groups and a large neutral or aromatic side group. It has been long thought that LAT1 is stereoselective, preferring only L-amino acids; however, recently, it has been proven that LAT1 can also carry some D-enantiomers [99,100]. LAT1 also operates with a rocking-bundle mechanism, in which the “bundle domain” goes through conformational changes from the outward-open state to the inward-open state (Figure 6) [14,15,101]. It is highly likely that this transport mechanism tolerates larger compounds than, e.g., the elevator-type mechanism used by EAATs and ASCTs.

**Figure 7:**
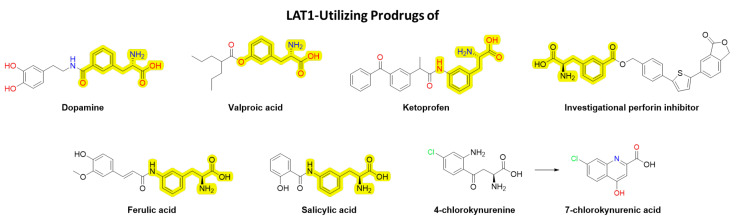
Molecular structures of prodrugs that can utilize LAT1 with promoieites highlighted with yellow color, excluding 4-chlorokynurenine, which undergoes internal cyclization to produce 7-chlorokynurenic acid.

Utilization of LAT1 for brain drug delivery has been proven not only by serendipitously discovered clinically used LAT1-substrates mentioned above, but also by rationally designed prodrugs, including drugs such as anti-inflammatories, antioxidants, antiepileptics, antiparkinsonians, immunosuppressants, and neuroprotective NMDA receptor antagonists (Figure 7) [102,103,104,105,106,107,108]. The greatest challenge with brain-targeted LAT1-utilizing prodrugs is to achieve the balance in bioconversion; not to have a too extensive premature release of the parent drug before crossing the BBB and, on the other hand, to gain an efficient bioconversion rate in the brain. The lack of knowledge on brain-specific prodrug bioconverting enzymes seems to be the greatest hurdle today; however, it is still an attainable goal. The crucial role of LAT1 in cancer has also increased the interest in developing LAT1-inhibitors as potential anticancer agents, such as JPH203, which has already advanced into clinical trials in Japan to treat solid tumors [109].

LAT2 and y+LAT2 have broader tissue distribution than LAT1, which makes brain-targeting of drugs more challenging (Table 3). LAT2 mediates sodium-independent efflux of many neutral amino acids (L-enantiomers of tyrosine, phenylalanine, tryptophan, threonine, asparagine, isoleucine, cysteine, serine, leucine, valine, glutamine, histidine, alanine, and methionine), while y+LAT2 mainly carries sodium-dependent exchange of L-arginine or L-leucine to L-glutamine (and Na^+^) [110,111,112]. LAT2 is also an exchanger with lower intracellular substrate affinities compared to extracellular substrate affinities, and similarly to LAT1, it functions with 1:1 stoichiometry [113]. LAT2 expression seems to be highest in microglia, although it is also expressed to some extent in the neurons and astrocytes (Figure 1d) [114]. Thus, this intra-brain selective expression could be utilized in microglia-targeted drug delivery of compounds. However, less is known about y+LAT2-specific localization in the brain, it has been reported to be expressed in the astrocytes and upregulated in the presence of ammonia (NH_4_^+^) [115,116].

Curiously, LAT2 can also carry thyroid hormones (T3 and 3,3′-diiodothyronine [117,118], the neurotransmitter precursor L-DOPA [119,120], although contribution to the overall transport of these compounds has been estimated to be minor compared to LAT1 and monocarboxylate transporters (MCT8/10). To date, no specific LAT2-inhibitor has been reported. LAT2 stimulates the mTOR pathway similarly to LAT1 and, thus, its overexpression has been reported in different types of cancers [120,121]. The transport mechanism of LAT2 and y+LAT2 resembles LAT1 and differs only with acidic and non-acidic residues in the binding pocket, which explains the substrate preferences of each transporter [122]. Thus, recent advances in structural and computational biology have enabled today’s successful design of LAT2- and y+LAT2-utilizing compounds. However, their minor expression in the brain compared to other transporters as well as wider distribution elsewhere in the body makes them less attractive carriers for brain-targeting purposes.

Asc-1 mediates a sodium-independent uptake of small amino acids, such as L-glycine, L-alanine, L-serine, L-threonine, L-cysteine, α-aminobutyric acid, and β-alanine, but it also carries D-isomers, such as D-serine [123,124]. Although Asc-1 facilitates the diffusion of small amino acids, it primarily functions as an exchanger (Table 3). Similarly, xCT is a sodium-independent exchanger of extracellular anionic cystine and intracellular glutamate (with a 1:1 stochiometry) [125,126]. Asc-1 is a neuronal transporter and due to its ability to carry L-glycine and D-serine in synapses, it has been considered a regulator of NMDA receptors [127,128]. Curiously, Asc-1 is mainly localized in those brain areas that are responsible for cognitive functions. Moreover, a lack of Acs-1 has been reported to cause tremors, ataxia, and seizures [129]. xCT has, in turn, been found in the brain from astrocytes and neurons, in addition to macrophages [130,131].

xCT functions together with EAATs, it counter-carriers the intracellular L-glutamine that EAATs have carried into the cells [132]. Similarly, it functions together with cysteine transporters, such as ASCTs, and counterbalances intracellular L-cysteine levels that xCT has provided to the cells. Therefore, xCT has a major role in health and diseases, e.g., downregulation of xCT impairs cell growth and survival. Furthermore, xCT mediates the protection of oxidative stress (cystine is a source of reduced glutathione, an endogenous antioxidant) and, therefore, it is upregulated in several different cancers [133,134]. Due to its role in both excitotoxicity (L-glutamine export) and oxidative stress (L-cysteine import), changes in xCT expression (both upregulation and downregulation) can have deleterious effects in the brain and, therefore, results in animal models of epilepsy and neurodegenerative diseases has been controversial [132]. Although several inhibitors have been developed for both of these transporters [135,136], less is known about the possibility of utilizing Acs-1 and xCT as drug carriers in the brain. Lack or minor expression of these transporters at the BBB, is the first challenge; however, their selective brain localization may offer some advantages in the intrabrain targeting. Therefore, more studies are definitely needed on these transporters in the future.

## 5. Monocarboxylate Transporter Family (SLC16A)

Monocarboxylate transporters (MCTs), members of the SLC16A family, facilitate rapid proton-dependent transport of monocarboxylates, such as lactate, pyruvate, and other metabolic products and energy substances in their anionic forms under physiological conditions [137]. The transportation across the cell membranes by MCTs is an electroneutral co-transport of monocarboxylates along with protons with a stoichiometric ratio of 1:1. The most important transporters of this family in the brain are MCT1 (*SLC16A1*), MCT2 (*SLC16A7*), MCT4 (*SLC16A3*), and MCT8 (*SLC16A2*) (Table 4), although other orphan MCTs, including MCT6 (*SLC16A5*), MCT7 (*SLC16A6*), MCT9 (*SLC16A9*), and MCT14 (*SLC16A14*) have also been found in the brain [138]. Ubiquitous MCT1 is expressed, e.g., in the epithelia of the small intestine and colon, but also in the muscles and particularly on the luminal and abluminal membranes of brain capillary endothelial cells [5,139]. In the brain, MCT1 is also expressed in the astrocytes of gray and white matter (Figure 1e) [140].

At the BBB, MCT1 has been proposed to be functioning bidirectionally to maintain brain homeostasis [141]. The efflux of compounds out of the brain back to the systemic circulation by MCT1 may also limit the brain drug disposition, like has been proposed in the case of probenecid and 6-mercaptopurine (Table 4) [142,143]. Curiously, a controlled substance γ-hydroxy butyric acid (GHB) that has been used clinically to treat insomnia, cataplexy, and narcolepsy is also an MCT1-substrate. Its CNS-related side effects, including respiratory depression, seizure, and loss of consciousness that may even result in coma and death, are predominantly arising from the variable MCT1-mediated transport of GHB across the BBB [144]. 4-Phenylbutyrate (4-PBA), a salt of an aromatic short-chain fatty acid and potential neuroprotective agent against excitotoxicity, oxidative stress, endoplasmic reticulum (ER) stress, apoptosis, and inflammation in several neurodegenerative disease models, such as PD, AD, and HD, has also been found to utilize MCT1 for its brain uptake [145]. MCT1 has also been proposed to be responsible for the transportation of salicylic acid, valproic acid, nicotinic acid, and some antibiotic β-lactams [146,147].

MCT8, in turn, is a specific thyroid hormone transporter mainly expressed in thyroid and adrenal glands, and other peripheral tissues, such as the liver and kidneys [148]. However, it is also highly expressed at the BBB and in neurons, and to some extent on the apical side of the choroid plexus [5,149,150]. MCT8 mediates the cellular uptake but also the efflux of thyroid hormones T3 and T4 (Table 4) [148,151], although the precise mechanism of their transport is still unknown. Studies in potential substrates and inhibitors of MCT8 suggest that the transporter is specific for the L-enantiomers of thyroid hormones. Amino and carboxyl groups of the alanine side-chain of thyroid hormones and at least one iodine atom in each iodothyronine ring are also required. Moreover, several drugs and natural compounds, including desipramine, dexamethasone, buspirone, desethylamiodarone, dronedarone, tyrosine kinase inhibitors, and silychristin have been reported to inhibit MCT8-mediated transport, either non-competitively or competitively [152].

The other members of the MCT family are expressed relatively selectively in the brain. MCT2 is the main MCT found in the neurons; it has been found on highly oxidative cells and cell bodies, including dendrites, dendritic spines, and axons of neurons, although there may be species differences (Figure 1e) [153,154]. MCT2 is also ubiquitously expressed in the peripheral tissues [155], making it challenging to utilize for intra-brain targeted drug delivery avoiding peripheral exposure. In turn, MCT3 (*SLC16A8*) is predominantly expressed in the choroid plexus epithelial cells, in addition to retinal epithelial cells [156], and MCT4 is predominantly expressed in astrocytes at least in rodents [153] (Table 4). It has been proposed that lipophilic statins, such as fluvastatin, atorvastatin, lovastatin, simvastatin, and cerivastatin in their acid form, can interact with MCT4 [157]. MCT4 is also known to be involved in the efflux transport of lactate in a pH-dependent manner and thus preventing its intracellular accumulation that could inhibit glycolysis in the cells [158]. Moreover, neurons can accumulate the effluxed lactate from astrocytes (cross-talk between MCT4 and MCT2) and utilize it as a secondary energy source [159].

Lactate is also highly produced in glycolytic cancer cells due to their higher energy demand, leading to intracellular acidification, unless removed from the cells. Therefore, MCT1, MCT2, and MCT4 are over-expressed in many different types of cancers and tumors, and, thus, targeting MCT-mediated lactate efflux could serve as a promising treatment or adjuvant therapy to other chemotherapeutics [160,161]. For example, an MCT1/2-selective inhibitor, AZD3965, has already been studied in phase I clinical trials against hematological cancers [162,163]. Furthermore, AZD3965 has been reported to be more effective than classical non-specific MCT inhibitor, 4-chloro-α-cyanocinnamic acid (α-CHCA) in the breast tumor model [164]. Curiously, MCT1 expression is increased at the BBB of children with attention-deficit/hyperactivity disorder (ADHD) [165], and expression changes of MCT1, 2, and 4 are related to metabolic states (obese and fasting individuals) in metabolic active tissues [166]. The expression of MCT1 is regulated mainly by transcriptional factors, such as MYC, and p53, while the expression of MCT2 has been associated with selective demethylation of an internal promoter region in the gene locus and reciprocal hyper-methylation of an upstream promoter region [167]. Contrarily, MCT4 is upregulated by hypoxia through a HIF-1α-dependent mechanism [141,168]. Mutations of MCT1 have also been reported to cause fatigue syndrome (muscle cramping and pain due to the impaired lactate removal after intense exercise) and MCT1 deficiency has been associated with recurrent ketoacidosis in children having a moderate intellectual disability, epilepsy, or migraine [166]. In turn, downregulation of MCT2 in the hippocampus and cerebral cortex has been associated with the pathologic progression of AD, possibly via reduced energy metabolism [169]. Furthermore, the absence of MCT3 expression in retinal pigment epithelium impairs visual functions and wound healing [170,171], and downregulation of MCT3 in smooth muscle cells via DNA methylation can induce the development of atherosclerosis [172]. A rare neurological disorder called Allan–Herndon–Dudley syndrome (AHDS) is a consequence of mutations in the MCT8 gene, leading to increased serum thyroid hormone levels and severe mental retardation and neurological dysfunctions [173,174,175]. In addition, inflammation has been reported to downregulate MCT8 [176]. Therefore, expression modulation of distinct MCTs in different diseases may have therapeutical potential.

**Figure 8:**
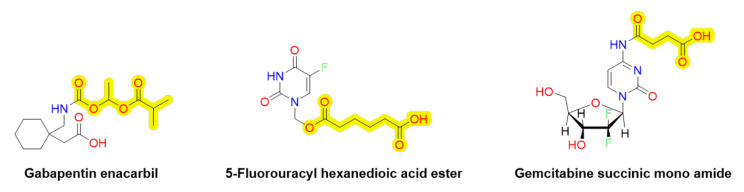
Molecular structures of prodrugs that can utilize MCT1 with promoieites highlighted with yellow color.

To date, MCT1-utilizing prodrugs have only been reported being used to improve oral bioavailability of compounds, such as 5-fluorouracil (5-FU), gemcitabine, and gabapentin by attaching mono-carboxylic acids with an ester or amide bonds (5-FU and gemcitabine), or shielding the free amino group with a polar acyloxyalkyl side chain with a carbamate bond (XP13512/GSK1838262, gabapentin enacarpil) (Figure 8) [177,178,179,180]. However, reports of utilizing MCTs for targeted or improved brain drug delivery via rationally designed prodrugs are less reported. Thus, it remains to be explored how well the reported MCT1-utilizing prodrugs of 5-FU and gemcitabine are transported into the brain, or into the specific brain cell types. Gabapentin enacarpil is already approved in the USA (Horizant^®^) and Japan (Regnite^®^) for the treatment of restless legs syndrome (RLS; moderate to severe) and the reported adverse effects have been related to CNS; most commonly severity, sedation, and dizziness [181]. Therefore, it is likely that gabapentin enacarpil has also improved brain drug delivery and exposure due to the increased MCT1-mediated transport (low affinity–high capacity transporter) across the BBB than its parent drug gabapentin, which is a LAT1-substrate (high affinity–low capacity transporter).

One challenge with MCTs is the bidirectional transport, which may be difficult to predict and control. Moreover, there are overlapping substrate specificities among MCTs. All the family SLC16A-members are known to have 12 transmembrane domains (TMDs) and an intracellular loop between TMDs 6 and 7. Furthermore, it has been proposed that MCTs facilitate a rocker switch transport mechanism, the same as GLUTs mediate (Figure 5) [64,182]. Structural-based analyses of the transport functions have provided some insights into the structure–activity relationship (SARs) of MCT1, MCT4, and MCT8 [183,184,185,186,187,188,189,190,191,192]. However, to develop successful MCT-utilizing prodrugs with improved brain drug delivery, more knowledge is required of the interactions that can affect the transport direction, particularly via MCT1 and MCT8 at the BBB, and MCT2 and MCT1/4 in neurons and astrocytes, respectively. Therefore, utilization of more sophisticated computational methods, such as molecular dynamics simulations (MDS) should be used in the future to reveal structural features of the prodrugs that can induce the conformational changes of the protein resulting in the translocation into the cells.

## 6. Organic Anion Transporting Family (SLCO/SLC21A)

The family of organic anion transporting polypeptides (OATPs) has had an inconsistent nomenclature in the past (OATP-A, OATP-B, OATP-C, etc.), and it was only in 2004 when this superfamily was standardized and renamed as an *SLCO*-family [193]. Another complicating factor with this family is that there are several gene duplicates and divergence between humans and rodents, e.g., OATP1A2 has five rodent orthologues, Oatp1a1, Oatp1a3, Oatps 1a4, Oatp1a5, and Oatp1a6; OATP1B1 and OATP1B3 have a single rodent orthologue (Oatp1a2); and OATP5A1 and OATP6A1 have three rodent orthologues (Oatp6b1, Oatp6c1, Oatp6d1), while other human OATPs have a name matching rodent orthologues [194]. Moreover, OATPs can be both ubiquitously distributed within the body, and very selectively expressed in certain tissues and there are species differences in these expression patterns. From the 11 members of this family that share approximately 40% amino acid sequence identity, at least OATP1A2 (*SLCO1A2*), OATP1C1 (*SLCO1C1*), OATP2A1 (*SLCO2A1*), OATP2B1 (*SLCO2B1*), and OATP3A1 (*SLCO3A1*) have been detected in the brain [194,195]. In addition, OATP4A1, OATP5A1, and OATP6A1 have been proposed to be expressed in the brain; however, these transporters are more or less orphans, i.e., whose functions are less well understood and, thus, these latter ones are not discussed in this review. More specifically, OATP1A2 and OATP2B1 are expressed at the BBB (luminal side), OATP1A2, OATP3A1 are neuronal transporters, OATP1C1 is an astrocytic transporter, and OATP2A1 is evenly distributed among neurons, astrocytes, and neurons (Figure 1f; Table 5) [5,196,197,198,199,200]. Curiously, OATP3A1 has two splice variants (OATP3A1_v1 and OATP3A1_v2) that are expressed in the brain in a dissimilar manner in neurons and choroid plexus.

Most of the OATPs transport a wide variety of compounds, both endogenous substrates, such as bile acids (e.g., cholate), steroids (e.g., estrone-3-sulfate), thyroid hormones (e.g., T3 and T4), and prostaglandins (e.g., PGE_2_), as well as exogenous substrates, such as hydroxymethylglutaryl-coenzyme A-CoA reductase inhibitors (statins), β-blockers (e.g., atenolol), and anticancer drugs (e.g., methotrexate), to name a few [194,195]. The main substrates of OATPs are anions; however, they can also carry neutral and cationic compounds (Table 5). In addition, many of their substrates are amphiphilic, having hydrophilic polar features and lipophilic proportions and they are relatively large (> 350 mol/g). In general, the substrate specificities of distinct OATPs overlap; however, some of them have also very narrow and precise substrate specificities. The transport via OATPs is sodium independent, but it can be affected by the pH. In an acidic environment, the transport activity, of at least some OATP subtypes, can be increased due to the increased substrate affinity via protonation of a conserved histidine residue at the extracellular end of TM3 [201]. The substrates of OATP1A2 and OATP2B1 overlap significantly with other OATPs as well as other transporters, and, therefore, specific structural features that would favor either OATP1A2- or OATP2B1-selective transport are relatively challenging to find [202,203]. A specific feature of OATP1C1 is the transport of thyroid hormones (T3, T4) and their derivatives [204]. Contrarily, OATP2A1 is known as a prostaglandin transporter, since it can transport, in addition to PGE_2_, several other prostaglandins (e.g., PGE_1_, PGE_3_, PGF_2α_, PGH_2_, and PGD_2_) that other OATPs are not able to carry, and it has a higher affinity for PGE_2_ compared to other OATP-subtypes [194,205]. Specific to OATP3A1 is the transport of arachidonic acid, and similar to OATP1C1 and OATP2A1, it has more discretesubstrate specificity and perhaps also narrowed to endogenous compounds [194,195]. However, it needs to be remembered that the lack of reported drug substrate and inhibitors may also be due to the limited number of studies that have been carried out with less studied OATP subtypes [206].

OATP1A2 is known to be inhibited by apple, orange, cranberry, and grapefruit juices that contain polyphenols and their conjugates, such as hesperidin, naringin, and avicularin (Table 5) [207,208]. In addition, rifampicin and verapamil as well as the third generation P-gylcoprotein (P-gp) inhibitors, elacridar, tariquidar, and zosuquidar, have been classified as OATP1A2 inhibitors [209,210]. Avicularin has also been reported to inhibit OATP2B1, which in turn, does not have selective inhibitors. However, many drugs and food additives have been reported to interfere with OATP2B1 function [211,212]. Therefore, both OATP1A2 and 2B1 are susceptible to drug–drug and drug–food interactions, already in the gastrointestinal tract, exemplified by well-known interactions with statins, fexofenadine (antihistamine), and aliskiren (renin inhibitor, used in the treatment of hypertension) [213,214]. Most likely, a similar interaction could occur also at the BBB. It has already been reported that prostaglandin transport via OATP2A1 is inhibited by polycyclic aromatic compounds, such as suramin (antiviral/antibacterial), pranlukast and zafirlukast (antiallergic/antiasthmatic), olmesartan and losartan (antihypertensive), and non-steroidal anti-inflammatories (NSAIDs; profens > anthranilates or fenamates), which can have huge effects on eicosanoid disposition [215]. Similarly, NSAIDs, particularly fenamates in addition to phenytoin (anti-seizure) can also inhibit OATP1C1-mediated transport and, thus, have a major impact on thyroid hormone brain disposition [204]. However, these compounds are more likely to be competing substrates rather than inhibitors of OATP1C1.

To date, 11 OATP1A2 single-nucleotide polymorphisms (SNPs) have been identified, some of them having reduced transport capacity of the substrates and some other SNPs having substrate-dependent changes in transport activity overall [216,217]. Therefore, OATP1A2 has a significant role in inter-individual differences in drug disposition in addition to drug–drug interactions (DDIs) (Table 5). Notably, it has been reported that the rodent orthologue Oatp1a4 is upregulated in hypoxia/reoxygenation via transforming growth factor-β (TGF-β)/activin receptor-like kinase 5 (ALK5) inhibition in the brain, which may offer an opportunity to optimize the CNS drug delivery, e.g., of statins [218]. For OATP2B1, only three missense SNPs have been identified to date, from which only one SNP has shown some effect on the transport capacity of selected probe drugs [219,220]. Since OATP1A2 and 2B1 carry many chemotherapeutics with relatively narrow therapeutic indices, the polymorphism of these OATPs can have dramatic effects on the efficacy and safety of these anti-cancer drugs. However, many OATPs, including 1A2 and 2B1, are overexpressed in many types of cancers, particularly in those which are highly dependent on the transport of steroid hormones required for cell proliferation, and thus affecting the exposure of their substrate drugs [221]. Curiously, OATP2A1 is downregulated in AD brain parenchymal cells [199], but upregulated in cancers, such as lung cancer via the PI3K/AKTmTOR pathway [222].

On the other hand, OATP1C1 has been reported to be downregulated together with another thyroid hormone transporter, MCT8, during the inflammation [176]. Moreover, Oatp1c1-deficiency and resulting hypothyroidism, have been proposed to cause neurological and behavioral alterations despite the presence of other thyroid hormone transporters, such as Mct8, in animal models [223,224]. Contrarily, OATP3A1 is upregulated in cholestasis (a liver disease where the flow of bile from the liver is reduced or blocked) via TNF-α-activated NF-κB-p65 and ERK-SP1 signaling pathways [225]. Overall, although OATP expressions are mainly regulated on the transcriptional level and as a response to their substrate levels, OATP localization and internalization from the plasma membrane can be affected by phosphorylation and preceding activation of protein kinases [226]. However, the expression modulation particular OATPs in selected conditions/diseases may have the potential to improve clinical outcomes, similar to MCTs.

OATPs have 12 TMDs with both termini located intracellularly. The large fifth extracellular loop has many conserved cysteine residues that can form disulfide bonds, and both the second and fifth extracellular loops contain several *N*-glycosylation sites [227]. It has been proposed that all OATP facilitate the translocation of their substrates through a positively charged pore in a rocker switch type of mechanism, similarly to GLUTs (Figure 5) [228]. Moreover, several amino acid residues that may have crucial roles in the OATP-mediated transport have been identified. However, due to the multiple binding sites of OATPs, the detailed interactions have remained controversial and, therefore, more efforts and additional computational experiments should be directed to understanding the exact translocation interactions of OATP substrates. This would also enable the rational design of OATP subtype-specific (pro)drug design and more efficient utilization of OATPs for brain drug delivery in the future, which has not been studied actively in the past. However, one great challenge with OATPs in brain drug delivery applications is their peripheral expression and overlapping substrate specificities, which may impair the targeting efficacy. Nevertheless, many of the already known substrates could be used as a starting point for the prodrug syntheses, e.g., thyroid hormone conjugates would be expected to be relatively specific for OATP1C1 among OATPs, but on the other hand, they could also be substrates of MCT8 and LAT1.

## 7. Organic Cation/Anion/Zwitterion Transporter Family (SLC22A)

The SLC22A family consists of organic cation transporters (OCTs) and organic anion transporters (OATs) carrying anions, cations, or zwitterions. OCT1 (*SLC22A1*), OCT2 (*SLC22A2*), and OCT3 (*SLC22A3*) are expressed most likely both at the luminal and abluminal sides of the BBB endothelial cells, although more evidence of the exact localizations is needed [229]. Furthermore, OCT2–3 has been identified in neurons and OCT2 in microglia and astrocytes (Table 6, Figure 1g). Similarly, OCTN1 (*SLC22A4*) and OCTN2 (*SLC22A5*) are most likely expressed at the BBB to some extent, but they are also found in parenchymal cells; OCTN1 in microglia and OCTN2 in neurons [229,230]. However, both OCTs and OCTNs are also expressed throughout the body, particularly in the kidneys and liver [194].

OCT1, OCT2, and OCT3 facilitate sodium and pH-independent transport of a broad range of endogenous and exogenous organic cations down their electro-chemical gradients, in both directions [194,231]. However, the affinity of OCT-ligands can depend on the degree of ionization, and therefore increased transport has been reported at lower pH [232]. Substrates of OCTs include a wide variety of structurally unrelated small organic cations for which they have different affinities (Table 6). These include commonly used probe-substrates, 1-methyl-4-phenylpyridinium (MPP) and tetraethylammonium (TEA), as well as endogenous compounds, such as choline, acetylcholine, dopamine, norepinephrine, epinephrine, serotonin, histamine, and agmatine, as well as drugs, such as quinidine, quinine, aciclovir, ganciclovir and metformin, amantadine, memantine, cimetidine, famotidine and ranitidine, cisplatin, debrisoquine, phenylcyclidine, clonidine, diphenylhydramine, atropine, procainamide, and cocaine, to name a few [231].

Noteworthy, the substrate and inhibitor specificities of OCT1, OCT2, and OCT3 overlap, and, therefore, some cations are transported by one OCT subtype and non-transported but bound (inhibitor) by another OCT subtype. Moreover, the degree and type of inhibition by a high concentration of a given inhibitory substrate may be total or partial [231]. In turn, the transport of OCTN1 and 2 can be sodium and pH-dependent or -independent, depending on the substrate. They are considered to be carnitine transporters; however, OCTN1 can selectively transport ergothioneine. OCTN1 and 2 are also known to carry, e.g., TEA, quinidine, pyrilamine, and verapamil [231].

OCT1 has been found to have 25 SNPs, while OCT3 has only five SNPs [233,234]. However, none of them are associated with human pathologies. Three SNPs of OCT1 and three SNPs of OCT3 are known to have reduced transport activity. Contrarily, OCT2 has ten transporter variants, which all are highly functional, but may have slightly altered substrate selectivity and transport capacity [231]. Nevertheless, the greatest risks with OCTs are related to DDIs, similar to OATPs [235]. Mutations in the OCTN gene, in turn, are directly linked to human autoimmune diseases; OCTN1 variant L503F is related to familial and sporadic inflammatory bowel disease, and multiple nonsense and missense mutations of OCTN2 are related to systemic carnitine deficiency [236,237].

From the OAT family, only OAT1 (*SLC22A6*) and OAT3 (*SLC22A8*) have been found in the brain, although they are highly expressed in the kidneys [194,238]. At the BBB, OAT3 is expressed on the abluminal side, but it has also a crucial role on the apical side of the choroid plexus, similarly to OAT1 (Table 6, Figure 1g) [239,240]. OATs function as anion exchangers and OAT3 has been proposed to participate in the efflux transport of organic anions, such as α-ketoglutarate, *para*-aminohippuric acid (PAH), benzylpenicillin, indoxyl sulfate, and homovanillic acid at the BBB (Table 6), carrying its substrates from the brain to blood. Although OAT1 and 3 can carry several endogenous and endogenous compounds, such as prostaglandins, non-steroidal anti-inflammatories (NSAIDs), antivirals, antibiotics (β-lactams), diuretics, antidiabetics, and anticancer drugs [241], the efflux transport direction makes them less suitable candidates for brain-targeted drug delivery. The uricosuric drug, probenecid, has been regarded as a potent inhibitor of OAT1 and 3, although it has also been referred to as OAT1/-3 substrate [194]. Therefore, with a concomitant administration of probenecid (or similar specific OAT1/-3 inhibitor), the OAT-mediated efflux of selected therapeutics (OAT1/-3 substrates) at the BBB could be avoided [242].

Due to the broad substrate specificity, OATs are related to several DDIs and currently, the U.S. Food and Drug Administration (FDA) and European Medicines Agency (EMA) recommend the evaluation of new chemical entities (NCEs) for their interactions with OAT1 and 3 [243,244]. For OAT1 and 3, several SNPs have been identified, some of which have decreased transporter function, while others do not have any effects [241]. However, due to the overlapping substrate specificities, other OAT members can replace the unfunctional transporter and, thus, mutations of OATs have been thought to have less significance for the clinical outcome.

OCTs and OATs have 12 TMDs with intracellular amino and carboxy-termini [194]. It has been shown that the extracellular loops between TMDs 1 and 2 are relatively large containing potential *N*-glycosylation sites, while the intracellular loops between 6 and 7 and the *C*-terminus have putative phosphorylation sites. These sites are prone to protein and tyrosine kinases and, therefore, activation of these kinases affects also the activity of the SLC22A-family transporters [245,246]. In addition, these transporters can be regulated at the transcriptional level. Since OCTs’ and OATs’ regulation varies a lot among the transporters, species, and tissue localization, more studies are warranted to better understand their life cycle.

Curiously, the exact transport mechanisms of the transporters in the SLC22A family are still unknown. Moreover, although OCTs and OATs are relatively well-explored transporters due to their crucial role in drug–drug interactions, less is known about their capability to carry drugs into the brain across the BBB, or inside the brain. Furthermore, the high hepato-renal expression of OCTs and OATs compared to the brain makes the brain-targeted drug delivery via them relatively challenging. However, since OCTs are responsible for carrying multiple drugs into the brain [238,247,248,249], and most likely more compounds will be discovered to utilize OCTs for their brain drug delivery, more efforts should be paid to the rational design of OCT-utilizing (pro)drugs in the future. Moreover, the overlapping substrate specificities may offer benefits of concomitant transport across the BBB and, thus, increase the overall brain drug disposition. Although, at the same time it will be at the expense of intra-brain selectivity, which is not likely to be achieved if a drug utilizes several OCTs. Nevertheless, all known drug carriers, including OATPs, OCTs, and OATs have the potential to be used as brain-targeted prodrug carriers. Many of their already known drug substrates could be used as starting point promoieties. Moreover, dual-drug targeting would have advantages in many complex neurodegenerative diseases, such as AD, since multiple targets could be hit with a double-prodrug at the same time. However, it should also be kept in mind that many natural substrates of other transporters, such as L-glutamine, L-cystine (GSH precursors), and L-arginine (precursors of L-ornithine and NO synthesis) that could be utilized as promoieties may also have additional beneficial effects in the brain.

## 8. Sodium-Coupled Neutral Amino Acid (System N/A) Transporter Family (SLC38A)

The family of SLC38 has eight sodium-coupled neutral amino acid transporters (SNATs) and three additional orphan transporters. From these SNAT1 (*SLC38A*), SNAT2 (*SLC38A2*), SNAT3 (*SLC38A3*), SNAT5 (*SLC38A5*), SNAT6 (*SLC38A6*), SNAT7 (*SLC38A7*), SNAT8 (*SLC38A8*), and thus excluding SNAT4 (*SLC38A4*), are expressed in the brain [250,251,252]. These transporters are further classified into system A and system N depending on their functional properties and patterns of substrate recognition. System A transporters (SNAT1 and SNAT2, also referred to as ATA1/SAT1 and ATA2/SAT2, respectively) recognize a broader range of aliphatic amino acids (L-proline, L-asparagine, L-cysteine, L-glutamine, L-glycine, L-methionine, and L-serine), whereas system N transporters (SNAT3 and SNAT5, also referred to as SN1 and SN2, respectively) have a rather narrow substrate profile (L-glutamine, L-histidine, and L-asparagine) (Table 7).

The expression of SNAT1 and SNAT2 is ubiquitous; however, they are also expressed in the brain, preferably in glutamatergic, GABAergic, and dopaminergic neurons but also to some extent in astrocytes (Figure 1h) [250,253,254,255,256]. In addition, SNAT2 has been found at the abluminal side of the BBB [5]. The transport of system A carriers (SNAT1 and 2) is sodium-dependent and highly pH-sensitive, and they exchange small and aliphatic amino acids for sodium with 1:1 stoichiometry. SNAT1 and 2 can be inhibited at low extracellular pH, but also by 2-methylamino-isobutyric acid (MeAIB), which is most likely a competing substrate of SNATs rather than an inhibitor (Table 7) [250,251].

System N transporters (SNAT3 and 5) are more tissue-specific, but in the brain, they have been suggested to be the major mediators of L-glutamine release from astrocytes (Table 7) and, thus, have a crucial role in the glutamate–GABA–glutamine cycle in the CNS together with system A transporters [250,257,258]. Distinctively, SNAT3, in addition to astrocytes, is also expressed in the luminal and abluminal sides of the BBB [5]. Similar to system A transporters, SNAT3 and 5 are also pH- and sodium-dependent; however, these transporters also accept Li^+^ substitution for Na^+^. Curiously, their transport activity can be enhanced by increasing the pH from 6 to 8 and they can function bidirectionally [258]. To date, no SNAT3-selective inhibitors have been reported, although glutamic acid-γ-hydroxamic acid (GluγHA) has been proposed as a SNAT5-selective inhibitor [259].

Transporters 6, 7, and 8 are yet unclassified according to the N/A-system and their role, particularly in the brain, is currently less well understood. SNAT6 is expressed exclusively in excitatory neurons, while SNAT7 and SNAT8 are expressed in the axons of the majority of neurons [260,261,262]. Therefore, these SNATs may also be important transporters in sustaining the glutamate neurotransmitter pool in the brain. It has been proposed that SNAT7 has similarities with system N, while SNAT8 resembles more of the system A. However, even less is known of SNAT6 function in relation to other SNATs [263].

At least SNAT1, SNAT2, and SNAT3 are upregulated in several different cancers, particularly in response to nutritional stress to compensate for the higher consumption of amino acids by the cancer cells [252]. In addition, SNAT2 has been found to be regulated by amino acids in neurons [256]. SNAT1 expression upregulation, in turn, has been linked to activated protein kinase A (PKA) signaling and SNAT1 expression downregulation to lipopolysaccharide-induced inflammation in astrocytes, but not in neurons (Table 7) [264,265]. The stable SNAT2 expression has been suggested to require an active mTOR-signaling, at least in placental trophoblast cells [266]. SNAT3 expression is regulated by insulin in hepatocytes; the calorie restriction upregulates its expression, while a chronic insulin treatment downregulates the expression via an mTOR pathway [267]. Moreover, increased protein kinase C (PKC) activity has been interlinked with SNAT3 internalization and expression downregulation [268]. SNAT5 expression regulation is less studied and understood.

The structure of SNATs is complex and heterogenic, although they all seem to contain 11 TMDs. SNAT1 has been proposed to have a long cytosolic *N*-terminus and a short extracellular *C*-terminus and a large glycosylated loop between TMD5 and 6, while SNAT2 most likely has an extracellular *C*-terminus and putative intracellular loops between TMD6 and 7 as well as 10 and 11 [250]. However, the transport mechanism of SNATs and how amino acids are involved in the pH-sensitivity of SNATs have remained unclear to date [252]. The SLC38A family has been structurally found to resemble a bacterial neurotransmitter-sodium symporter, LeuT and a proton-dependent amino acid transport ApcT, which facilitate the rocking bundle mechanism, similarly to LAT1 (Figure 6) [251]. Therefore, it is highly likely that SNATs could mediate the rocking bundle mechanism; however, more detailed insights into the mechanism from higher resolution structures and MDS studies are required. They are also highly needed to enable rational SNAT-utilizing (pro)drug design and to reveal the transport direction. It would be crucial to understand how structural features can affect the transport direction, particularly at the BBB to ensure efficient SNAT-mediated delivery to the target sites. Moreover, with SNATs, there is a great potential to achieve intrabrain selective drug delivery, by utilizing system A transporters for neuronal accumulation and system N carriers for astrocytal accumulation.

## 9. Other Transporters in Discrete Families

Vitamin transporters that belong to different families, such as sodium-dependent multivitamin transporter, SMVT (*SLC5A6*) and sodium-vitamin C co-transporter 2, SVCT2 (*SLC23A2*), are also highly expressed in the brain. SMVT has been primarily found at the BBB while SVCT2 is more of a neuronal transporter; however, it has also been found in reactive astrocytes [269,270,271]. Some attempts to utilize these transporters for targeted drug delivery have been reported in the past, but overall, only little is known of the interactions of these transporters and their substrates. Well-known substrates of SMVT include biotin, pantothenic acid, and lipoic acid, while only L-ascorbic acid (AA) has been reported as an SVCT2 substrate [272,273]. Notably, the oxidized form, dehydroascorbic acid (DHA), is not only an SVCT2-substrate, it has also been proposed to be transported via GLUT1 and GLUT3. Therefore, it has been suggested that SVCT2 is a part of the AA recycling mechanism within the brain, where DHA is uptake at the BBB and by astrocytes via GLUT1, reduced to AA and released from the astrocytes, and re-uptaken into the neurons via SVCT2 [270,271,273]. Although both transporters are expressed in peripheral tissues too, only SVCT2 has been utilized for brain-targeted purposes by using a prodrug technology. NSAIDs, such as naproxen and ibuprofen, have been conjugated with AA and glucose, and shown improved brain accumulation [274,275,276]. However, their transport mechanism remains to be solved, whether it is mediated via GLUT1 or SVCT2. So far, SMVT has been utilized only to improve the oral bioavailability and topical ocular delivery of antivirals, saquinavir, and acyclovir [277,278], and their delivery into the brain has remained unknown.

In the SLC5A-family, there is also a choline transporter, CHT (*SLC5A7*), that has a crucial role in the brain, particularly in cholinergic neurons [279,280,281]. However, CHT is not the only choline transporter, also OCT1–3 (discussed above) and choline transporter-like proteins 1–5 (CTL1–5, *SLC44A1–5*) participate in choline homeostasis. From the latter ones at least, CTL1, CLT2, and CTL5 have been found in the brain, CTL1 and CLT2, particularly at the BBB, but CLT1 also in neurons and glial cells [279,282,283]. Unfortunately, CHT and CLTs and their roles in the brain are not yet well understood. It has been proposed that they may be involved in the pathogenesis of neurodegenerative diseases, such as AD, and, therefore, their expression modulation could have therapeutical potential [282]. Nevertheless, more studies are also needed to reveal if these transporters could be harnessed for rational prodrug design. Their function is known to be inhibited by several cationic drugs [279], and, therefore, it is likely that they could carry also drugs, in addition to choline.

Curiously, members of the SLC2A-family (GLUTs) are not the only glucose carriers, also sodium-glucose cotransporters, SGLTs (*SLC5A1–12*), have been recognized as sodium-dependent unidirectional sugar carriers [47,49]. From these carriers, at least SGLT1 and 2 have been detected in the brain, although also with smaller expression compared to GLUT1 and 3. Therefore, it is not likely that higher brain drug disposition can be achieved by utilizing the SGLT-transporters; however, in the cases where GLUTs are downregulated, like in AD, additional glucose transporters may have a more critical role in the transport of gluco-conjugates. Some inhibitors of SGLTs (gluco-conjugates and others) have been reported to maintain stable plasma glucose in type 2 diabetic patients, mainly due to the inhibition in the intestine and renal proximal tubules [284,285,286]. Nevertheless, more structural and functional details overall are required from all the above-mentioned transporters in order to deploy them for rational (pro)drug design in brain-targeted applications.

In addition, there are also several nucleotide transporters expressed in the brain, such as sodium-independent equilibrative nucleoside transporters 1–4 (ENT1–4, *SLC29A1–4*) and to a lesser extent sodium-dependent concentrative nucleoside transporters 1–3 (CNT1–3; *SLC28A1–3*) [287,288,289,290]. These transporters can carry not only endogenous nucleosides but also synthetic nucleoside analogs, including anticancer drugs such as 5-fluorouracil, 6-mercaptopurine, cladribine, gemcitabine, fludarabine, and cytarabine, antiviral drugs, such as ribavirin, zalcitabine, and zidovudine. Therefore, purine and pyrimidine nucleosides could be used as promoieties in the prodrug design. Thus, there are also other possibilities and several transporters that could be utilized in the brain-targeted transporter-utilizing prodrug approach in the future.

## 10. Conclusions and Future Prospects

Targeting drugs into the brain or improving the brain drug delivery to the therapeutically relevant levels has remained an unanswerable challenge until today. However, we have come a long way from thinking that increased lipophilicity would increase the passive permeation across the BBB, although, there is still a huge task to increase our understanding of the roles of dozens or hundreds of orphan transporters and to utilize transporters more broadly in therapeutical applications [291,292,293,294]. Unfortunately, in science, we tend to focus on hot topics; thus, only a limited number of transporters are very well characterized for different purposes. However, it is hoped that this review encourages particularly young scientists to explore the transporter field even more broadly and without any prejudice in the future.

As presented above, there are several different transporters expressed at the BBB and/or selectively expressed in brain parenchymal cells that are relatively well-characterized and to some extent also utilized for brain-targeted and intrabrain-targeted drug delivery, respectively. Some of the brain-delivered therapeutics are drugs acting as such; however, in many cases a prodrug approach is needed to be utilized in order to temporarily change the pharmacokinetic properties of potent drugs. This requires not only selective transporter-mediated cellular uptake but also a biotransformation step to release the active parent drug, which in many cases, has been the most challenging part of brain-targeted prodrug applications. Therefore, there are not so many clinically used brain-targeted prodrugs available these days despite the success of the prodrug approach for other purposes, such as increasing the solubility of oral absorption [11,12,13]. L-Dopa, a LAT1-substrate, is one of the few examples as it undergoes enzymatic decarboxylation to release dopamine.

The majority of prodrugs have hydrolyzable bonds, such as ester bonds, which predisposes the prodrugs to premature first-pass metabolism before they reach the brain. On the other hand, more stable amide prodrug bonds have not released their parent drugs at a sufficient rate in the brain. Moreover, transporters are not the only ones with a great expression variety among the species as well as between the patients. Additionally, enzyme expression levels can vary, which complicates not only the translation from preclinical data to the clinical situation, but also affects the efficacy and safety of the treatments of a particular patient. Compared to transporters, even less is known about the roles of different enzymes in the brain. As mentioned many times in this review, computational methods should be utilized more extensively to understand the exact translocation mechanisms of substrates and transport direction across the transporter cavities, which would increase our success in drug development. However, computational methods should also be used more extensively to design extended-release prodrugs and to find binding and mechanistic catalytic differences among hydrolyzing enzymes in the brain vs. peripheral tissues.

Overall, more efforts should be paid to the brain drug delivery of already used marketed compounds to increase our understanding. Curiously, statins (OATP-substrates) are cyclized lactones that are hydrolyzed to their acid form and they have effects also in the brain [295]. Thus, they could be considered brain-delivered prodrugs. In addition, many OCT-substrates seem to have effects in the brain as such (not prodrugs), including memantine, fluoxetine, and ketamine [229]. Therefore, in addition to already extensively studied LAT1, OATPs and OCTs could be utilized for brain drug delivery in the future. Of course, with these suggested transporters, the targeting efficacy into the brain can be a challenge due to their expression in other tissues. On the other hand, LAT1 successfully delivers L-dopa into the brain, although it is also expressed in many other peripheral tissues. Thus, the expression intensities in different tissues have a great impact on targeting potential. Based on this fact, GLUT1, CAT1, and MCT1 with high BBB expression profiles, should also be considered suitable brain-targeted carriers in the future.

Nevertheless, it needs to be remembered that carrier-mediated drug delivery is only one option and most likely, it is not suitable for all therapeutical agents. Biological drugs are becoming more and more common, and as macromolecules, they are most likely to be delivered into the brain via other routes, like receptor-mediated transport. In addition, other administration routes, such as trans-nasal administration, are still under extensive research. Therefore, more efforts should be paid also to those delivery mechanisms and how they could be utilized more effectively for the brain-targeted as well as intrabrain-targeted purposes.

It is obvious that there will never be 100% brain-targeting avoiding other tissues or cells completely by any developed delivery method, since transporters and receptors are also expressed in other tissues and cells. However, if we can increase the concentration at the target site to the therapeutically relevant level and simultaneously decrease the concentrations in off-target cells, it may have an enormous impact on the clinical outcome. Nevertheless, the protein expression intensity and localization is not the only determinant when targeting compounds, the overlapping substrate specificities of transporters and receptors have also a huge impact on the compounds’ distributions. It is more than likely that a single compound is able to utilize more than one transport mechanism. Therefore, if the target transporter or receptor expression or function is downregulated due to the disease condition or polymorphism, there can be additional carriers or mechanisms that can completely change the pharmacokinetic profile of the selected compound. This can have both, negative and targeting minimizing effects, as well as positive and targeting increasing effects. Therefore, more efforts should be paid to secondary interactions with other proteins in the future, in order to obtain successful CNS drugs for clinical use.

Thus, to summarize, in brain-targeted approaches, it is all about finding the right balance. In a transporter-utilizing prodrug approach, it would be a balance among transporter expression profiles and tissue-specific localization, a balance with transporter selectivity and substrate specificities, and a balance between bioconversion rate in peripheral tissues and at the CNS to gain appropriate delivery and targeting of pharmacologically active compounds.

## Figures and Tables

**Table 1:** Tissue distribution, substrates, inhibitors, and expression/function modulators of EAAT1–3 and ASCT1–2.

Transporter	Gene Name	Tissue Distribution (Expression)	Substrates	Inhibitors	Expression Modulation/Transport Capacity Changes
EAAT1	*SLC1A3*	Brain: BBB (abluminal), astrocytes	L-Glu, L-Asp	L-Serine-*O*-sulfate (L-SOS), (*R,S*)-2-amino- 3-(1-hydroxy-1,2,3-triazol-5-yl)propionate, (4*R*)-4-methylglutamate (4-Me-Glu), UCPH-101, UCPH-102	EAAT1 expression ↑ by adenylate cyclase- activating polypeptide (PACAP), transforming growth factor α (TGFα), epidermal growth factor (EGF), estrogen, tamoxifen, raloxifen
EAAT2	*SLC1A2*	Brain: BBB (abluminal), astrocytes	L-Glu, L-Asp	Dihydrokainic acid, WAY-213613	EAAT2 expression ↑ by adenylate cyclase- activating polypeptide (PACAP), transforming growth factor α (TGFα), epidermal growth factor (EGF), estrogen, tamoxifen, raloxifen, glucocorticoids, ceftriaxone
EAAT3	*SLC1A1*	Brain: neurons	L-Glu, L-Asp	2-(Furan-2-yl)- 8-methyl-N-(o-tolyl)imidazo[1,2-a]pyridin-3-amine	Amphetamine induces EAAT3 internalization
ASCT1	*SLC1A4*	Ubiquitous, Brain: luminal and abluminal membranes of BBB, neurons, and astrocytes	L-Ala, L-Ser, L-Cys, L-Gly, L-Met, L-Val, L-Leu, L-Ile, L-Thr, D-Ser; L-Glu (efflux)	Phenylglycine analogs	ASCT1 expression ↓ results in neurodevelopmental alterations
ASCT2	*SLC1A5*	Ubiquitous, Brain: BBB (abluminal), neurons, and astrocytes	L-Ala, L-Ser, L-Gly, L-Met, L-Val, L-Leu, L-Ile, L-Thr; L-Glu (efflux)	*O*-Benzyl- L-serine, *S*-benzyl- cysteine, phenylglycine analogs	ASCT2 expression ↑ in highly proliferative cells, such as cancer cells

↑ (arrow up) represents upregulation of the protein; ↓ (arrow down) represents downregulation of the protein.

**Table 2:** Tissue distribution, substrates, inhibitors, and expression/function modulators of GLUT1 and GLUT3.

Transporter	Gene Name	Tissue Distribution (Expression)	Substrates	Inhibitors	Expression Modulation/Transport Capacity Changes
GLUT1	*SLC2A1*	Ubiquitous, Brain: luminal and abluminal membranes of BBB, astrocytes, (neurons, microglia)	Glucose, galactose, mannose, 2-deoxy-D-glucose, 2-deoxy-2-[^18^F]-D-glucose, glucosamine and dehydroascorbic acid (vitamin C)	Cytochalasin B, forskolin, phloretin and other flavonoids, WZB117, BAY-876, STF-31, fasentin, apigenin	GLUT1 expression ↑ in numerous cancers and ischemia with poor survival of patients: via hypoxia, p53, PI3K-Akt pathways, Ras or c-Myc oncogenes; GLUT1 expression ↓ in Alzheimer’s disease and GLUT1 deficiency syndrome (G1DS) due to mutations
GLUT3	*SLC2A3*	Brain: neurons	D-Glucose, D-galactose, D-mannose, D-xylose, 2-deoxy-D-glucose	Cytochalasin B, forskolin, phloretin, quercetin and other flavonoids, glycogen synthase kinase-3 (GSK-3) inhibitors	GLUT3 expression ↑ in various cancers with poor survival of patients: via hypoxia, p53, PI3K-Akt pathway

↑ (arrow up) represents upregulation of the protein; ↓ (arrow down) represents downregulation of the protein.

**Table 3:** Tissue distribution, substrates, inhibitors, and expression/function modulators of CAT1–3 and LAT1–2, y+LAT2, Asc-1, and xCT.

Transporter	Gene Name	Tissue Distribution (Expression)	Substrates	Inhibitors	Expression Modulation/Transport Capacity Changes
CAT1	*SLC7A1*	Ubiquitous, Brain: luminal and abluminal membranes of BBB	L-Arg, L-Lys, and L-Orn	Not known	CAT1 expression ↓ via NMDA receptor activation; CAT1 expression ↑ in colorectal and breast cancers, hepatitis B virus-induced hepatocellular carcinoma, and lymphocytic leukemia
CAT2B	*SLC7A2*	Brain: neurons, oligo-dendrocytes, induced astrocytes	L-Arg, L-Lys, and L-Orn	Not known	CAT2B expression ↑ in different breast cancer cell lines
CAT3	*SLC7A3*	Placenta, Brain: neurons	L-Arg, L-Lys, and L-Orn	Not known	CAT3 expression ↓ via NMDA receptor activation:
LAT1	*SLC7A5*	Widely distributed, Brain: luminal and abluminal membranes of BBB, neurons, astrocytes, microglia	L-Leu, L-Phe, L-Tyr, L-Trp, L-His, L-Met, L-Ile, L-Val; triiodothyronine (T3) and thyroxine (T4), L-dopa, melphalan, baclofen, gabapentin, pregabalin	JPH203 (unselective)	LAT1 expression ↑ in numerous cancers with poor survival of patients: via hypoxia/HIF-2α, c-Myc or RAS-MEK-ERK pathways
y+LAT2	*SLC7A6*	Ubiquitous, Brain: astrocytes	L-Arg, L-Leu, L-glu (efflux)	No specific inhibitor reported	y+LAT2 expression ↑ in the presence of NH4^+^: via NF-κB pathway
LAT2	*SLC7A8*	Ubiquitous, Brain: microglia > neurons > astrocytes	L-Tyr, L-Phe, L-Trp, L-Thr, L-Asn, L-Ile, L-Cys, L-Ser, L-Leu, L-Val, L-Gln, L-His, L-Ala, L-Met; triiodothyronine (T3), 3,3′-diiodothyronine	No specific inhibitor reported	LAT2 expression ↑ in highly proliferative cells, such as cancer cells
Asc-1	*SLC7A10*	Adipose tissue, Brain: neurons	L-glycine, L-alanine, D-/L-serine, L-threonine, L-cysteine, α-aminobutyric acid, and β-alanine	Several structures have been proposed, requires more studies	Asc-1 downregulation associated with tremors, ataxia, and seizures
xCT	*SLC7A11*	Macrophages, Brain: astrocytes, neurons	Cystine (extracellular)/glutamate (intracellular) exchange	*S*-4-carboxy-3-hydroxy- phenylglycine, erastatin, sorafenib, sulfasalazine	xCT is upregulated in several cancers and its dysfunction is associated with epileptic seizures, neurodegeneration, and brain edema

↑ (arrow up) represents upregulation of the protein; ↓ (arrow down) represents downregulation of the protein.

**Table 4:** Tissue distribution, substrates, inhibitors, and expression/function modulators of MCT1–4 and MCT8.

Transporter	Gene Name	Tissue Distribution (Expression)	Substrates	Inhibitors	Expression Modulation/Transport Capacity Changes
MCT1	*SLC16A1*	Ubiquitous, Brain: luminal and abluminal membranes of BBB, astrocytes	Lactate, pyruvate, ketone bodies; probenecid, 6-mercapto-purine, 4-phenyl-butyrate salicylic acid, nicotinic acid, valproic acid, β-lactams, XP13512, γ-hydroxy butyric acid	4-Chloro-α-cyanocinnamic acid (non-specific), AZD3965	MCT1 expression ↑ in numerous cancers, at the BBB of ADHD children, and metabolic active tissues of obese individuals: via MYC, p53
MCT2	*SLC16A7*	Liver, kidneys, Brain: neurons	Lactate, pyruvate, ketone bodies	4-Chloro-α-cyanocinnamic acid (non-specific), AZD3965	MCT2 expression ↑ in numerous cancers and metabolic active tissues of obese individuals: via demethylation and hyper-methylation of DNA; MCT2 expression ↓ in hippocampus and cerebral cortex with pathologic progression of Alzheimer’s disease (via reduced energy metabolism?)
MCT3	*SLC16A8*	Retinal pigment epithelium, choroid plexus	Lactate	Not reported	MCT3 expression ↓ in retinal pigment epithelium impairs visual functions and wound healing and in smooth muscle cells induces atherosclerosis via DNA methylation
MCT4	*SLC16A3*	Skeletal muscles, intestine, kidneys, heart, Brain: astrocytes	Lactate, pyruvate, ketone bodies; fluvastatin, atorvastatin, lovastatin, simvastatin, cerivastatin in their acid form	4-Chloro-α-cyanocinnamic acid (non-specific)	MCT4 expression ↑ in numerous cancers and in muscles of obese individuals: via hypoxia/HIF-1α
MCT8	*SLC16A2*	Liver, endocrine tissues, Brain: luminal and abluminal membranes of BBB, neurons	Thyroid hormones (T3 and T4)	Possibly desipramine, dexa-methasone, buspirone, desethyl- amiodarone, dronedarone, tyrosine kinase inhibitors, and silychristin	MCT8 expression ↓ in Allan–Herndon–Dudley syndrome and during the inflammation

↑ (arrow up) represents upregulation of the protein; ↓ (arrow down) represents downregulation of the protein.

**Table 5:** Tissue distribution, substrates, inhibitors, and expression/function modulators of selected members of the OATP-family.

Transporter	Gene Name	Tissue Distribution (Expression)	Substrates	Inhibitors	Expression Modulation/Transport Capacity Changes
OATP1A2	*SLCO1A2*	Ubiquitous, Brain: luminal membranes of BBB, neurons	Anionic, cationic, and neutral amphiphilic compounds of large size with different affinities among the transporters Endogenous substrates, such as bile acids (cholate), steroids (estrone-3-sulfate), thyroid hormones (T3 and T4), prosta-glandins (PGE_2_); Exogenous substrates, such as statins (fluvastatin), β-blockers (atenolol), and anticancer drugs (methotrexate)	Fruit juices that contain polyphenols and their conjugates, such as hesperidin, naringin, and avicularin; rifampicin, verapamil, elacridar, tariquidar, and zosuquidar (possibly competing substrates)	11 single-nucleotide polymorphisms (SNPs): transport activity ↓ (substrate-specific); OATP1A2 expression ↑ in several cancers: possible involvement of hypoxia/reoxygenation in the upregulation at the BBB
OATP1C1	*SLCO1C1*	Testis Brain: astrocytes, choroid plexus		NSAIDs, (fenamates), phenytoin (competing substrates exhibiting mutual inhibition function)	OATP1C1 expression ↓ during the inflammation
OATP2A1	*SLCO2A1*	Ubiquitous, Brain: neurons, astrocytes, and microglia		Polycyclic aromatic compounds, such as suramin, pranlukast, zafirlukast, olmesartan, losartan, non-steroidal anti-inflammatories	OATP2A1 expression ↓ in AD brain parenchymal cells; OATP2A1 expression ↑ in cancers: via PI3K/AKT/mTOR pathway
OATP2B1	*SLCO2B1*	Ubiquitous, Brain: luminal membranes of BBB		Some of the substrates are also reported as inhibitors due to the drug–drug interactions	11 single-nucleotide polymorphisms (SNPs): transport activity ↓ with 6 SNPs OATP2B1 expression ↑ in several cancers
OATP3A1	*SLCO3A1*	Ubiquitous, Brain: neurons			OATP3A1 expression ↑ in cholestasis: via TNF-α-activated NF-κB-p65 and ERK-SP1 signaling

↑ (arrow up) represents upregulation of the protein; ↓ (arrow down) represents downregulation of the protein.

**Table 6:** Tissue distribution, substrates, inhibitors, and expression/function modulators of OCT1–3, OCTN1–2, and OAT1–3.

Transporter	Gene Name	Tissue Distribution (Expression)	Substrates	Inhibitors	Expression Modulation/Transport Capacity Changes
OCT1	*SLC22A1*	Liver and other peripheral tissues, Brain: BBB	Organic cations with different affinities among the transporters Endogenous substrates: catecholamines, monoamine neurotransmitters Drugs: several cytostatic, antiviral, antibiotic, antioxidant, psycho-stimulant, anti-hypertensive, antiemetic, antidepressan, antidiabetic agents	Transported substrates of the OCTs exhibit mutual inhibition function	18 single-nucleotide polymorphisms (SNPs): transport activity ↓ with 6 SNPs See more information below (OATs)
OCT2	*SLC22A2*	Kidneys and other peripheral tissues, Brain: BBB, neurons, microglia, astrocytes		Cytochalasin B, forskolin, phloretin, quercetin and other flavonoids, glycogensynthase kinase-3 (GSK-3) inhibitors	10 transporter variants with altered substrate selectivity and transport capacity
OCT3	*SLC22A3*	Abundant, Brain: BBB, neurons			5 SNPs: transport activity ↓ with 3 SNPs
OCTN1	*SLC22A4*	Abundant, Brain: BBB, microglia	Acetylcholine, ergothioneine, L-carnitine, TEA, quinidine, pyrilamine, and verapamil	Transported substrates of the OCTNs exhibit mutual inhibition function	OCTN1 variant L503F: familial/ sporadic inflammatory bowel disease
OCTN2	*SLC22A5*	Abundant, Brain: BBB, neurons	Acetyl-L- carnitine, D-/L-carnitine, TEA, quinidine, pyrilamine, and verapamil	Transported substrates of the OCTNs exhibit mutual inhibition function	Multiple OCTN2 variants: systemic carnitine deficiency
OAT1	*SLC22A6*	Kidneys, Brain: choroid plexus	Overlapping substrate specificities, although not identical, transports several drugs Endogenous substrates: α-ketoglutarate, *para*-amino-hippuric acid, benzoyl penicillins, indoxyl sulfate, and homovanillic acid, and prostaglandins	Probenecid (inhibitor/ substrate)	Multiple SNPs: decreased function can be compensated with other transporters in the family due to the overlapping substrate specificities; NOTE! OCTs and OATs are all regulated in transcriptional level as well as by post-translational phosphorylation via several protein kinases
OAT3	*SLC22A8*	Kidneys, Brain: abluminal side of the BBB, choroid plexus			

↓ (arrow down) represents downregulation of the protein.

**Table 7:** Tissue distribution, substrates, inhibitors, and expression/function modulators of SNAT1–2 (system A) and SNAT3 and 5 (system N).

Transporter	Gene Name	Tissue Distribution (Expression)	Substrates	Inhibitors	Expression Modulation/Transport Capacity Changes
SNAT1	*SLC38A1*	Ubiquitous, Brain: neurons (astrocytes)	L-proline, L-asparagine, L-cysteine, L-glutamine, L-glycine, L-methionine, and L-serine	2-Methylamino-isobutyric acid (MeAIB, competing substrate); low pH	SNAT1 expression ↑: via protein kinase A (PKA) activation; SNAT1 expression ↓: via inflammation; SNAT1 expression ↑ in many types of cancers
SNAT2	*SLC38A2*	Ubiquitous, Brain: abluminal side of the BBB, neurons (astrocytes)	L-proline, L-asparagine, L-cysteine, L-glutamine, L-glycine, L-methionine, and L-serine	2-Methylamino-isobutyric acid (MeAIB; competing substrate); low pH	Stable SNAT2 expression requires an active mTOR-signaling; SNAT2 expression ↑ in many types of cancers
SNAT3	*SLC38A3*	Liver, kidney, muscles, eye, Brain: luminal and abluminal sides of the BBB, astrocytes	L-glutamine, L-histidine, and L-asparagine	Not reported	SNAT3 expression ↓ by insulin: via an mTOR pathway; SNAT3 expression ↑ by calorie restriction: via increased protein kinase C (PKC) activity(?); SNAT3 expression ↑ in many types of cancers
SNAT5	*SLC38A5*	Intestinal tract, kidney, retina, lung, Brain: astrocytes	L-glutamine, L-histidine, and L-asparagine	Glutamic acid-γ-hydroxamic acid (GluγHA)	Less studied

↑ (arrow up) represents upregulation of the protein; ↓ (arrow down) represents downregulation of the protein.

## Data Availability

Not applicable.
